# A LDH‐Based Supramolecular Photosensitizer‐Functionalized Bioactive Glass Scaffold for Integrated Postoperative Osteosarcoma Recurrence Prevention and Bone Regeneration

**DOI:** 10.1002/advs.202524296

**Published:** 2026-02-09

**Authors:** Tao Wang, Yixin Bian, Tingting Hu, Mengyang Li, Yuqin Tan, Yu Yang, Xiaolan Jian, Xisheng Weng, Chaoliang Tan, Ruizheng Liang

**Affiliations:** ^1^ State Key Laboratory of Chemical Resource Engineering Beijing Advanced Innovation Center for Soft Matter Science and Engineering Beijing University of Chemical Technology Beijing P. R. China; ^2^ Department of Orthopedic Surgery State Key Laboratory of Complex Severe and Rare Diseases Peking Union Medical College Hospital Chinese Academy of Medical Science and Peking Union Medical College Beijing P. R. China; ^3^ Department of Electrical Engineering & Department of Biomedical Engineering City University of Hong Kong Kowloon Tong Hong Kong SAR P. R. China; ^4^ Graduate School Hunan University of Chinese Medicine Changsha Hunan P. R. China; ^5^ Hunan Provincial Hospital of Integrated Traditional Chinese and Western Medicine Changsha Hunan P. R. China; ^6^ Hong Kong Branch of National Precious Metals Material Engineering Research Center (NPMM) City University of Hong Kong Kowloon Tong Hong Kong SAR P. R. China; ^7^ Shenzhen Research Institute City University of Hong Kong Shenzhen P. R. China; ^8^ Quzhou Institute For Innovation in Resource Chemical Engineering Quzhou P. R. China

**Keywords:** bioactive glass scaffolds, bone regeneration, layered double hydroxides, osteosarcoma treatment, photodynamic therapy

## Abstract

Layered double hydroxide (LDH)‐based materials have been explored as efficient photosensitizers for photodynamic therapy (PDT) or functional agents for bone regeneration, holding great promise in the synergistic PDT‐mediated osteosarcoma treatment and bone reconstruction. Herein, we report the functionalization of 5‐iodoisophthalic acid (I‐IPA)‐intercalated MgZnAl‐LDH onto bioactive glass scaffold (BGS) to construct a bifunctional composite scaffold (BGS/I‐LDH) for integrated osteosarcoma treatment and bone regeneration. The surface functionalization of I‐LDH onto BGS confers BGS/I‐LDH high‐efficiency reactive oxygen species generation performance under 1270 nm laser irradiation, resulting in a singlet oxygen quantum yield up to 1.53, which is significantly superior to that of previously reported NIR‐excited PSs. Moreover, thanks to the sustained release of Mg^2+^ and Zn^2+^ ions from I‐LDH, BGS/I‐LDH exhibits exceptional anti‐osteosarcoma and osteogenic properties with 3.8‐fold bone volume, 3.0‐fold bone mineral density, and 11.4‐fold new bone mass increments compared with the pristine BGS, which is remarkably more preponderant than other previously reported modified BGS.

## Graphical abstract

An intercalation assembly strategy is proposed to construct a novel LDH‐based supramolecular photosensitizer, which exhibits outstanding reactive oxygen species generation activity under 1270 nm laser irradiation. The deposition of supramolecular photosensitizer onto the surface of bioactive glass scaffolds results in the development of an innovative dual‐functional scaffold for the management of osteosarcoma and promotion of bone regeneration.

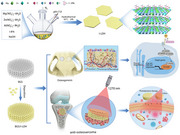

## 1 Introduction

Osteosarcoma is an invasive malignant bone tumor that predominantly affects adolescents, manifested by ostealgia, structural deformities, and functional impairments [1, 2]. Current clinical treatment of osteosarcoma primarily relies on surgical resection and perioperative adjuvant chemotherapy [3, 4]. However, incomplete surgical resection, side effects of chemotherapy, and high invasiveness of the tumor usually result in tumor recurrence and metastasis [5, 6]. Additionally, osteosarcoma inevitably leads to bone defects, seriously affecting postoperative functional reconstruction [7, 8]. Therefore, optimal treatment of osteosarcoma should strive to combine thorough residual tumor cell elimination with effective bone structural reconstruction. To date, several alternatives have been proposed that combine osteogenic nanoplatforms involving various scaffold materials such as magnesium (Mg) alloy, chitosan, hydrogel, polycaprolactone (PCL), hydroxyapatite (HAp), and bioactive glass scaffolds (BGS) with photothermal, photodynamic, chemodynamic, magnetothermal, chemotherapeutic agents, as well as immune stimulants and gene expression regulators [9, 10]. Among them, the BGS with favorable biocompatibility, mechanical properties, and osteogenic characteristics has been widely exploited in bone regeneration. The ability of bioactive glass to form a strong bond with host tissue, stimulate new bone formation, and gradually degrade over time to be replaced by natural bone makes it particularly suitable for bone tissue engineering. For comparison, hydrogels have excellent flexibility and can mimic the extracellular matrix, making them suitable for cell encapsulation and drug delivery systems [11, 12]. However, their mechanical strength and ability to support bone formation may be limited. PCL is known for its biodegradability and mechanical properties [13, 14], but it may lack the bioactive properties needed to promote bone growth and integration. Therefore, BGS stands out for its unique combination of bioactivity, osteoconductivity, and mechanical properties, making it a promising material for osteosarcoma treatment and bone regeneration, which can be easily functionalized for extended application scenarios, representing a potential alternative for integrating bone reconstruction with tumor treatment [15]. For instance, Wang et al. impregnated active single‐atom iron catalysts onto BGS to develop a nanocomposite with significant photothermal ablation of osteosarcoma and bone tissue regeneration capabilities [16]. Yang et al. reinforced BGS with black phosphorus to achieve photothermal ablation of osteosarcoma and subsequent phosphorus‐driven biomineralization [17]. However, the osteogenic performance of the above composite scaffolds mainly stems from BGS itself, whose insufficient osteogenic activity leads to suboptimal bone regeneration and remodeling. Thus, it is necessary to develop a novel functionalized BGS with enhanced osteogenic properties and anti‐tumor function for comprehensive postoperative osteosarcoma management and efficient critical‐sized bone defect reconstruction.

Layered double hydroxides (LDHs), as a typical kind of layered nanomaterials characterized by exceptional biocompatibility, adjustable chemical composition and physical structure [18, 19, 20, 21, 22, 23, 24, 25], have been demonstrated to possess immense potential in tumor diagnosis [26, 27, 28, 29], cancer treatment [30, 31, 32], drug delivery [33, 34], and tissue engineering [36, 37, 38, 39]. For example, isophthalic acid/ZnAl‐LDH nanohybrids (IPA/LDH) constructed by an intercalation confinement strategy could act as a supramolecular photosensitizer (PS) with a singlet oxygen (^1^O_2_) quantum yield of 0.74 for two‐photon NIR‐I photodynamic therapy (PDT) [40]. Owing to the deep tissue penetration depth and large maximum permissible exposure of NIR‐II, ultrathin CoMo‐LDH and CoCuMo‐LDH nanosheets activated by defect engineering were proposed as highly active inorganic PSs for NIR‐II PDT to efficiently kill tumor cells in vitro and ablate tumors in vivo [41, 42]. Recently, the surface functionalization of MgAlEu‐LDH on HAp scaffold was reported [43], which not only significantly improved the specific surface, surface roughness, and hydrophilicity of HAp scaffold for promoting cell adhesion and osteogenic differentiation, but also sustainedly released Mg^2+^ and Eu^3+^ ions for vascular regeneration and bone repair. Despite the considerable promise of LDH‐based materials in bone regeneration or tumor treatment, synergistic cancer therapy/bone repair has been rarely reported and is still in its infancy [44, 45, 46, 47]. In addition, there is significant room for improvement in the synergistic anti‐tumor effect and osteogenic performance of LDH‐based materials. Therefore, the development of LDH‐based composite scaffolds with significantly enhanced therapeutic efficacy against osteosarcoma and outstanding osteogenic properties is promising and of great significance for the prevention of postoperative osteosarcoma recurrence and skeletal reconstruction.

**FIGURE 1:**
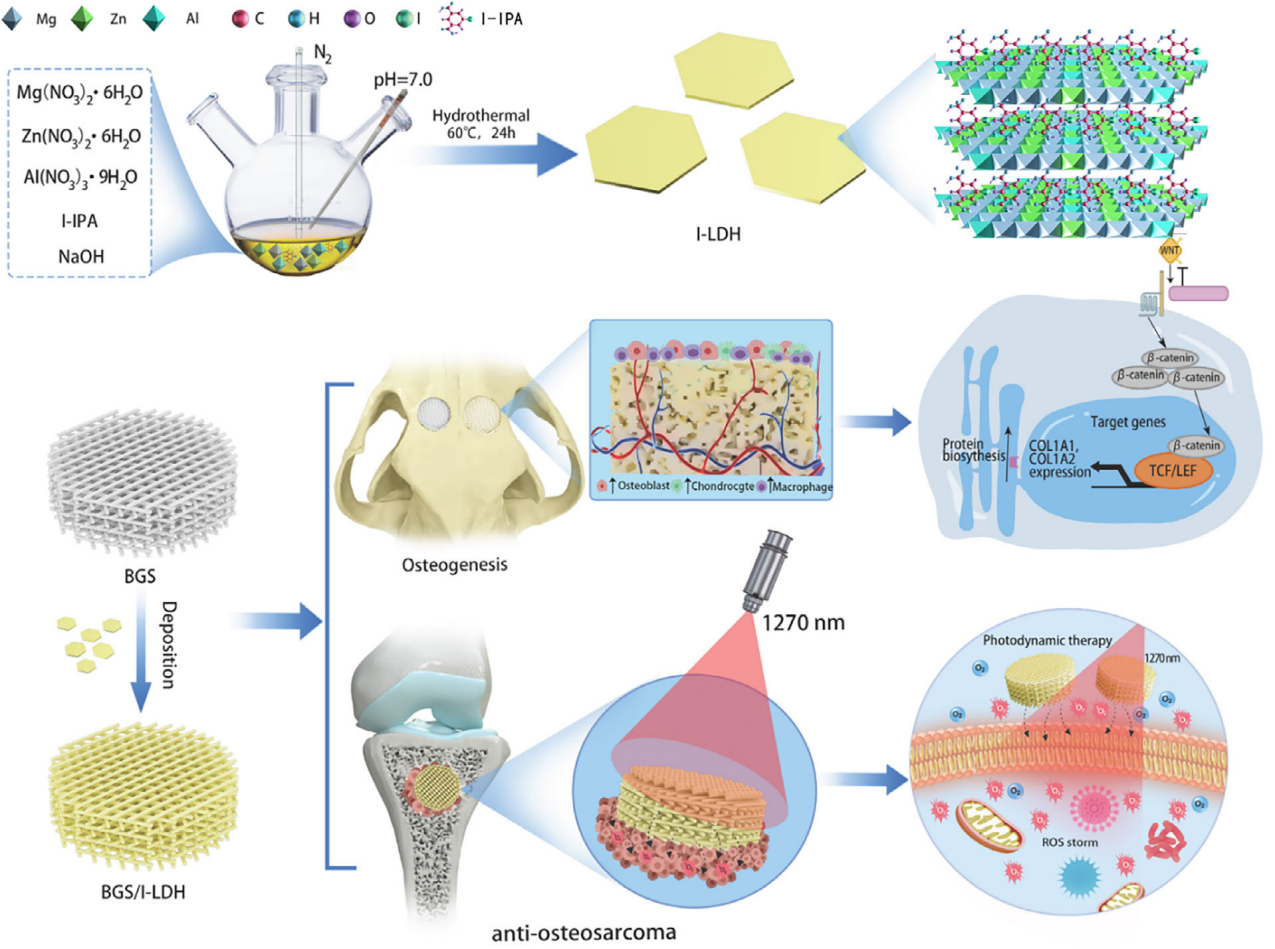
Schematic illustration of BGS/I‐LDH preparation and its application in synergistic PDT‐mediated osteosarcoma management and bone regeneration.

As an emerging tumor treatment modality, PDT has aroused increasing attention because of its noninvasiveness, high spatiotemporal precision, and limited side effects, which employs PSs to generate reactive oxygen species (ROS) under light irradiation for killing cancer cells [48, 49, 50, 51, 52, 53, 54, 55, 56, 57]. Herein, we report the design and preparation of an innovative bifunctional scaffold (BGS/I‐LDH) by deposition of 5‐iodo‐isophthalic acid (I‐IPA)‐intercalated MgZnAl‐LDH (I‐LDH) on BGS for synergistic PDT‐mediated osteosarcoma management and bone regeneration (Figure 1). Specifically, the I‐LDH exhibits high‐efficiency PDT performance under 1270 nm laser irradiation due to the enhanced ROS generation mediated by confinement effect, resulting in a ^1^O_2_ quantum yield up to 1.53, which is significantly superior to that of other previously reported NIR‐excited PSs. Importantly, I‐LDH functionalized BGS scaffold enables the sustained release of Mg^2+^ and Zn^2+^ ions with excellent osteogenic properties from LDH within two weeks, supporting long‐term bone repair. Moreover, in vivo experiments verify the exceptional photodynamic effect and osteogenic properties of BGS/I‐LDH, which achieves efficient osteosarcoma treatment without postoperative recurrence and comprehensive cranial defect reconstruction with 3.8‐fold bone volume, 3.0‐fold bone mineral density, and 11.4‐fold new bone mass increments when compared to BGS, remarkably preponderant than other previously reported modified BGS. Transcriptome sequencing analysis and further molecular verification unveil the activation of the typical osteogenic Wnt/β‐catenin/COL1 signaling pathway as the underlying mechanism for the osteogenesis processes of BGS/I‐LDH. Single‐cell sequencing further demonstrates a remarkable increment in osteoblasts, macrophages, and chondrocytes around BGS/I‐LDH compared with pristine BGS. Our study provides a dual‐functional scaffold (BGS/I‐LDH) with outstanding capabilities in the efficient treatment of osteosarcoma, prevention of postoperative recurrence, and promotion of bone regeneration, which holds significant promise for clinical application.

## 2 Results and Discussion

### 2.1 Preparation and Characterization of I‐LDH

**FIGURE 2:**
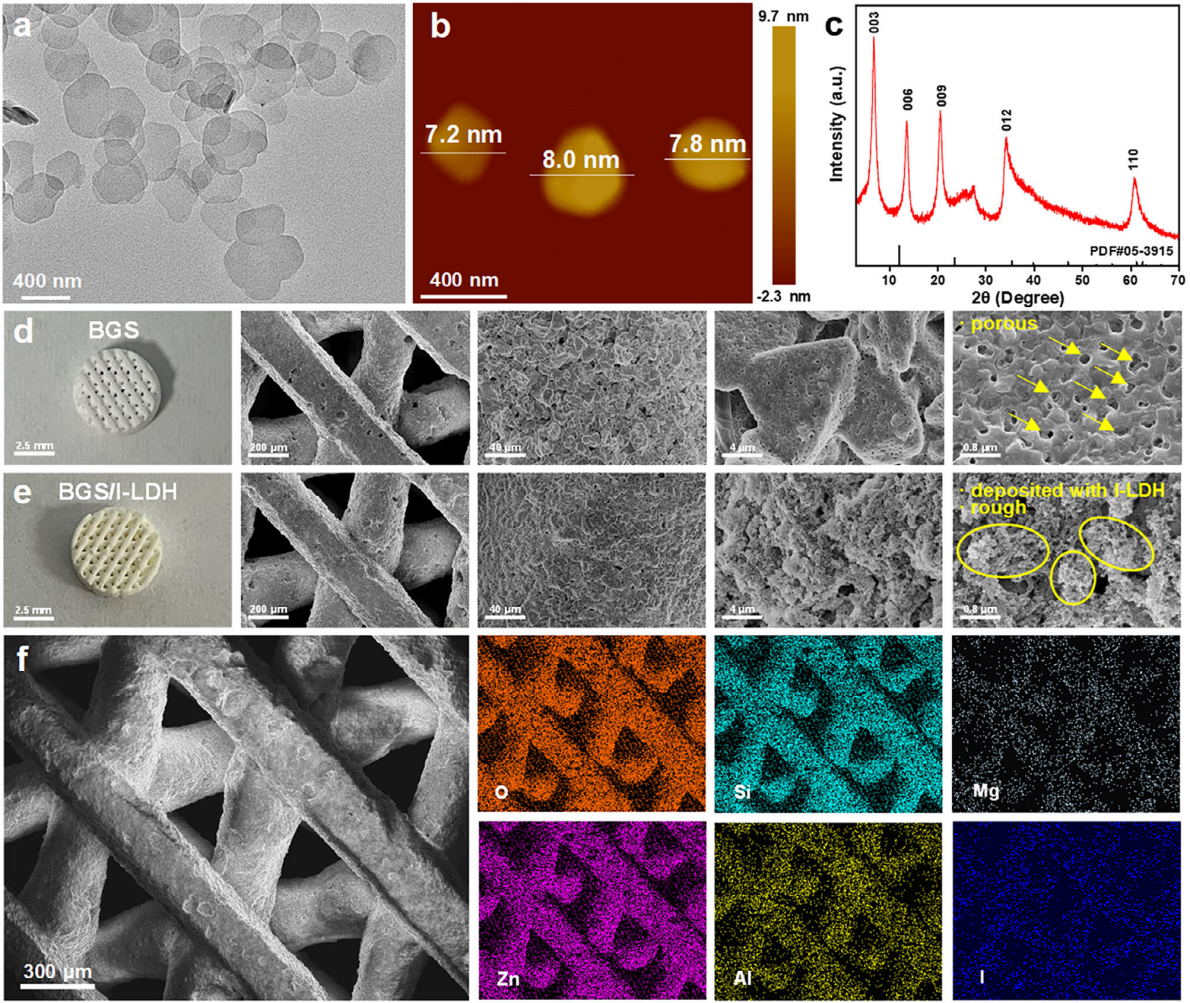
(a) TEM image, (b) AFM image, and (c) XRD pattern of I‐LDH nanosheets. SEM images of (d) BGS and (e) BGS/I‐LDH scaffolds. (f) EDX mapping images of the BGS/I‐LDH scaffold.

The I‐LDH was obtained by intercalating I‐IPA into the interlayers of layered MgZnAl‐LDH host using a classical coprecipitation method. The transmission electron microscopy (TEM) image exhibits the uniform nanosheet morphology of I‐LDH with an average diameter of 450 ± 11 nm (Figure 2a). A thickness of 7.2–8.0 nm for I‐LDH was revealed by atomic force microscopy (AFM) (Figure 2b; Figure S1). As observed from the X‐ray diffraction (XRD) patterns in Figure 2c and Figure S2, both MgZnAl‐LDH and I‐LDH exhibit obvious characteristic diffraction peaks at the (003), (006), and (009) planes of the LDH crystal. Compared with MgZnAl‐LDH, the diffraction peaks in the XRD pattern of I‐LDH shift to a lower angle, suggesting the successful intercalation of I‐IPA into MgZnAl‐LDH. Fourier‐transform Infrared (FT‐IR) spectra display the absorption peaks (700 and 770 cm^−1^) of the meta‐substitution of the benzene ring and the characteristic bands of LDH at 1354 cm^−1^ (vibration absorption peak of C = O in CO_3_^2−^) for I‐LDH (Figure S3a), confirming the intercalation of I‐IPA into MgZnAl‐LDH. In addition, the Zeta potential of MgZnAl‐LDH in water decreases significantly from 47.1 ± 1.5 mV to 8.44 ± 2.2 mV after combination with I‐IPA (Figure S3b). According to the UV‐vis‐NIR absorption spectrum (Figure S4), I‐LDH has strong absorption in the NIR‐II region (1100–2000 nm), establishing a prerequisite for NIR‐II PDT. The molar ratios of the main elements in I‐LDH were measured using inductively coupled plasma‐mass spectrometry (ICP‐MS), as shown in Table S1.

### 2.2 Synthesis and Characterization of BGS/I‐LDH Scaffolds

The BGS/I‐LDH composites were constructed by integrating I‐LDH nanosheets into the BGS via a surface coating method. Digital photos showed that the surface of the BGS/I‐LDH was yellowing while the pristine BGS was white (Figure 2d,e). The surface micromorphology was then investigated by scanning electron microscope (SEM). As observed in Figure 2d, the BGS has a loose porous structure on the surface, while the surface of BGS/I‐LDH is much rougher (Figure 2e), which is conducive to cell adhesion. The Mercury Intrusion Porosimetry (MIP) test results showed that compared with BGS, the porosity and specific surface area of BGS/I‐LDH increased, while the average pore diameter decreased significantly, effectively benefiting bone ingrowth and promoting nutrient exchange (Figure S5). Elemental mapping images demonstrated the uniform distribution of O, Si (all from BGS), Mg, Zn, Al, and I (all from I‐LDH) elements on BGS/I‐LDH (Figure 2f). ICP‐MS analysis also detected several main elements originating from the I‐LDH in the BGS/I‐LDH (Table S2). The mechanical properties of scaffolds before and after functionalization of I‐LDH were measured by an electronic universal testing machine. As presented in Figure S6, the compression strength and compressive modulus of BGS/I‐LDH were 1.2 times and 2.1 times that of the BGS, demonstrating that I‐LDH functionalization improved the compressive capacity of the BGS.

To evaluate the in vitro degradability of the composite materials, BGS and BGS/I‐LDH scaffolds were immersed in PBS solutions with pH values of 5.4, 6.5, and 7.4, and assessed over 8 weeks. Quantitative analysis showed that the degradation rate of both scaffolds decreased as the pH increased. BGS/I‐LDH consistently exhibited a slightly higher mass loss than BGS under all pH conditions, which may be attributed to the relatively high dissolution of I‐LDH coating compared to the BGS matrix (Figure S7). Further in vivo degradability of BGS and BGS/I‐LDH scaffolds was assessed by subcutaneously implanting the scaffolds into Sprague–Dawley rats over the same 8‐week period. Quantitative analysis revealed that BGS retained over 93% of its original mass, indicating minimal degradation over 8 weeks. In comparison, BGS/I‐LDH showed a slightly higher weight loss of approximately 13%, likely due to the degradation of the I‐LDH component (Figure S8). These findings suggest that the incorporation of I‐LDH slightly increases the degradability of the whole scaffold.

### 2.3 Detection of ^1^O_2_ Generation of I‐LDH

**FIGURE 3:**
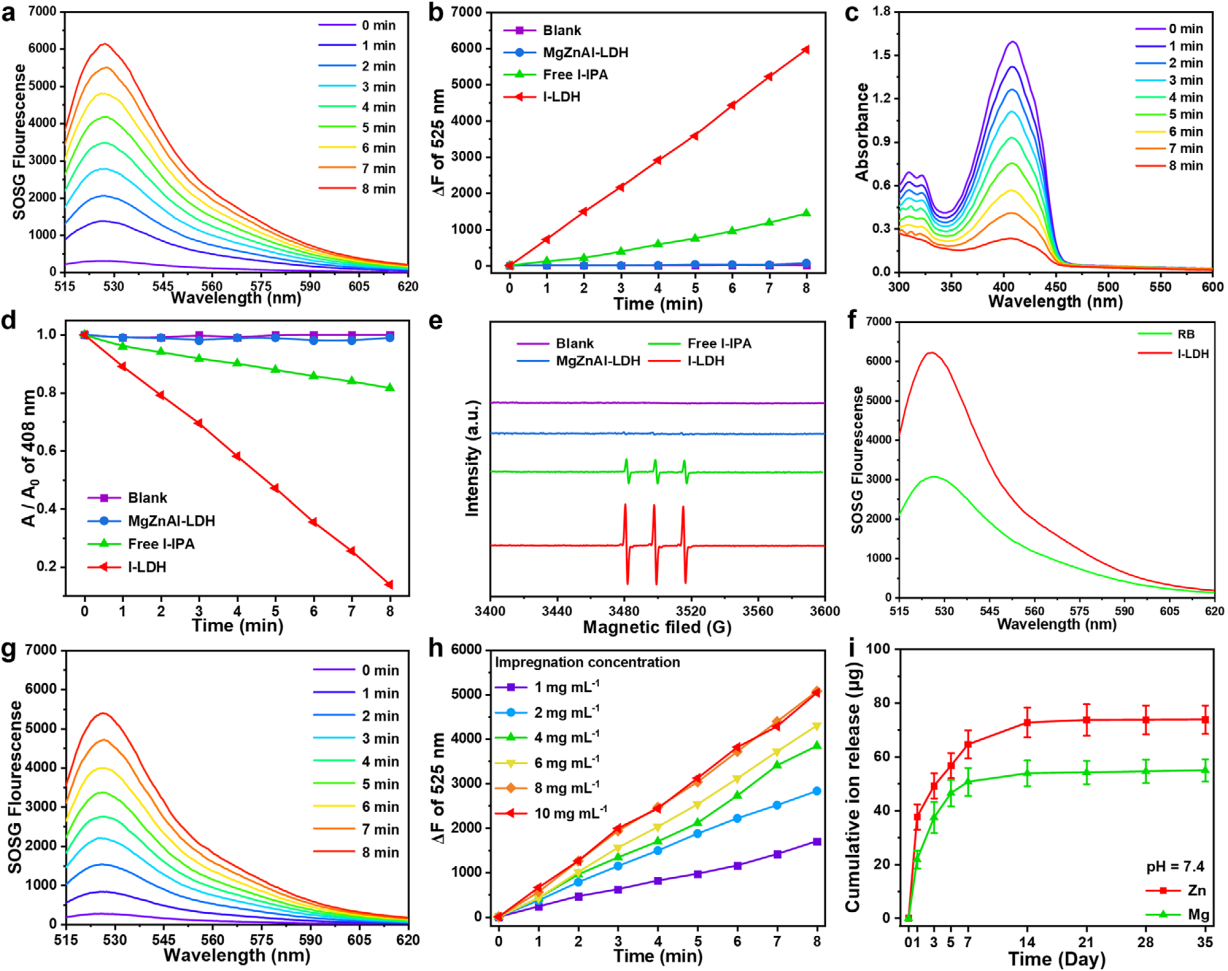
(a) Fluorescence spectra of SOSG in the presence of I‐LDH in H_2_O under 1270 nm laser irradiation (0.75 W cm^−2^). (b) Fluorescence intensity of SOSG as a function of irradiation time. (c) UV–vis spectra of DPBF in the presence of I‐LDH in H_2_O under 1270 nm laser irradiation (0.75 W cm^−2^). (d) Normalized absorbance decay of DPBF as a function of irradiation time. (e) ESR spectra of TEMP/^1^O_2_ for blank, MgZnAl‐LDH, free I‐IPA, and I‐LDH after 1270 nm laser irradiation (0.75 W cm^−2^, 8 min). (f) Fluorescence intensity of SOSG in the presence of I‐LDH after 1270 nm laser irradiation (0.75 W cm^−2^, 8 min) and RB after 550 nm laser irradiation (0.75 W cm^−2^, 8 min). (g) Fluorescence spectra of SOSG in the presence of BGS/I‐LDH scaffold in H_2_O under 1270 nm laser irradiation (0.75 W cm^−2^). (h) Fluorescence intensity of SOSG in the presence of BGS/I‐LDH scaffolds at different impregnation concentrations. (i) Zn and Mg ions release kinetics of BGS/I‐LDH scaffolds under simulated environments at pH 7.4. Data are presented as mean values ± s.d. (*n* = 3).

The ^1^O_2_ generation ability of I‐LDH under 1270 nm (0.75 W cm^−2^) irradiation was evaluated using a singlet oxygen sensor green (SOSG) fluorescence probe [58]. As exhibited in Figure S9, no increase in fluorescence intensity of SOSG under 1270 nm laser irradiation was observed in MgZnAl‐LDH, and a slight increase in fluorescence intensity of SOSG was found in the free I‐IPA group. In contrast, the fluorescence intensity of SOSG in I‐LDH (100 µg mL^−1^) solution increased violently during the 8 min irradiation (Figure 3a,b), indicating that the confined triplet exciton can significantly improve the ^1^O_2_ generation capacity of I‐LDH. Typically, the generation of ^1^O_2_ was also examined by 1,3‐diphenylisobenzofuran (DPBF) assay. As a control, no decrease in DPBF absorbance was observed in the blank and MgZnAl‐LDH groups. After 1270 nm laser irradiation, the free I‐IPA produced a small amount of ^1^O_2_, leading to a slight decrease in DPBF absorbance (Figure S10). By contrast, a rapid decrease in the absorbance of DPBF was observed in the I‐LDH group (Figure 3c,d) upon 1270 nm laser irradiation, confirming the excellent ^1^O_2_ generation capacity of I‐LDH. Interestingly, the typical 1:1:1 triplet pattern of ^1^O_2_ was detected in the electron spin resonance (ESR) spectrum of free I‐IPA under 1270 nm laser irradiation (Figure 3e), which was significantly enhanced in the I‐LDH group. In addition, the ESR signal exhibited an irradiation time‐dependent enhancement (Figure S11). The ^1^O_2_ quantum yield of I‐LDH was calculated to be 1.53 using Rose Bengal (RB) as a reference PS (Figure 3f; Figure S12), which is significantly superior to that of other previously reported NIR‐excited PSs (Table S3). Such a high ^1^O_2_ quantum yield can ensure the efficient photodynamic osteosarcoma treatment.

### 2.4 Detection of ^1^O_2_ Generation and Ion Release of BGS/I‐LDH Scaffolds

The as‐designed BGS/I‐LDH also displayed excellent photodynamic activity on account of the high ^1^O_2_ quantum yield ability of I‐LDH. As shown in Figure 3g, the elevation of SOSG fluorescence intensity at 525 nm suggested the generation of ^1^O_2_ by BGS/I‐LDH upon 1270 nm laser irradiation. The fluorescence intensity of SOSG significantly increased with the augmentation of the impregnation concentration and reached a peak level at 8 mg mL^−1^ impregnation (Figure 3h; Figure S13). The results of DPBF tests were consistent with those of SOSG (Figure S14). The ion release kinetics of BGS/I‐LDH composite scaffolds are presented in Figure 3i and Figure S15. At pH 7.4, Zn^2+^ and Mg^2+^ displayed rapid release within the first week, reaching a plateau by the second week. Reducing the pH to 6.5 and 5.4 markedly accelerated ion release rates. At pH 5.4, equilibrium was attained within one week with elevated cumulative release. These findings confirm that the scaffold can release Zn^2+^/Mg^2+^ within the bone tumor resection site (pH 6.0–7.0) to enhance osteogenesis, while sustained ion release ensures the prolonged bone remodeling [59, 60, 61, 62].

### 2.5 In Vitro Evaluation of the Biocompatibility, Anti‐Osteosarcoma Effect, and Osteogenic Properties

**FIGURE 4:**
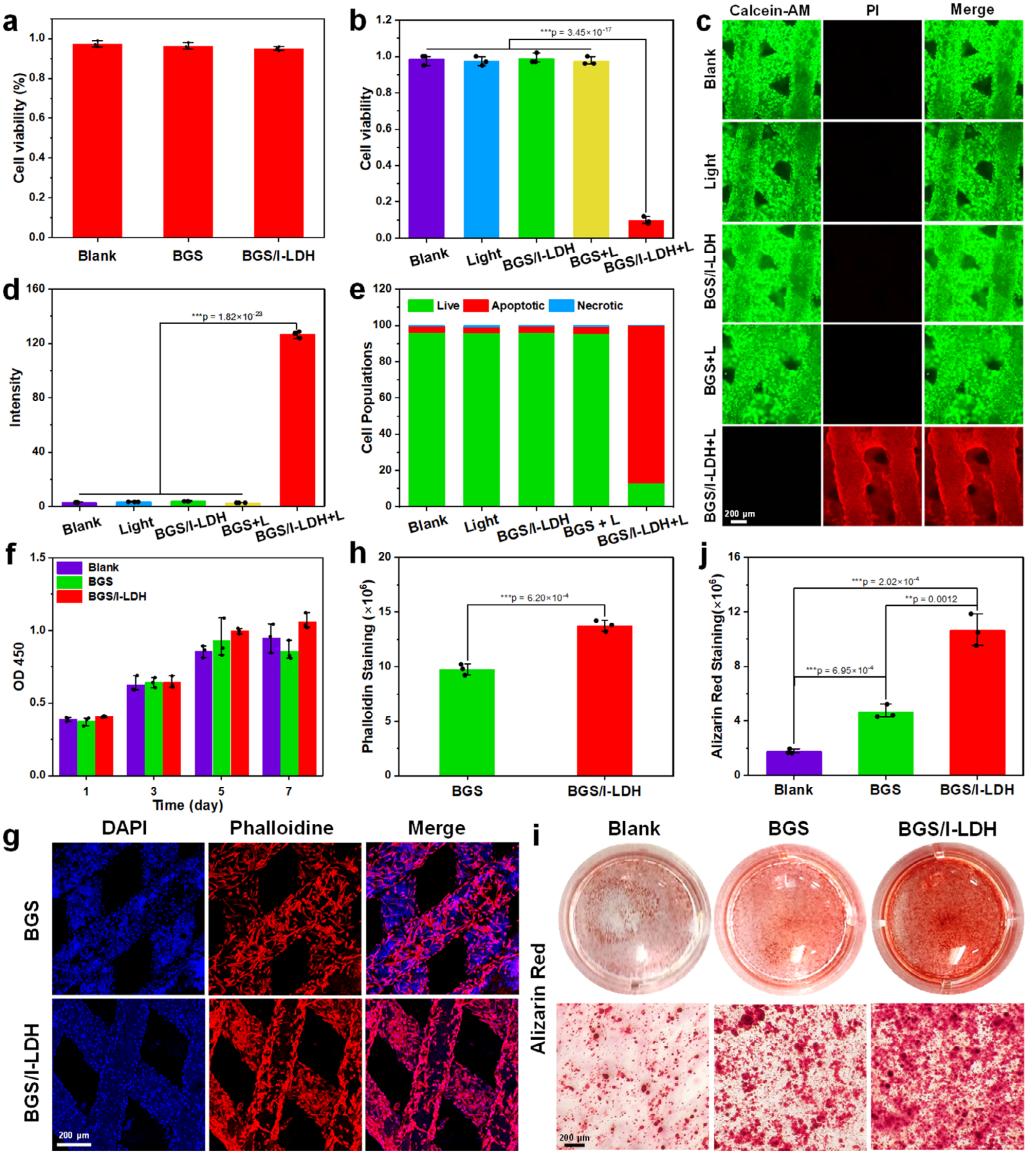
(a) MTT assay of the viability of MC3T3‐E1 cells incubated with BGS or BGS/I‐LDH scaffolds. (b) In vitro cellular cytotoxicity profiles of Saos‐2 cells after different treatments: blank, light (0.75 W cm^−2^, 6 min), BGS/I‐LDH, BGS + L, and BGS/I‐LDH + L, and (c) corresponding Calcein‐AM/PI staining images. (d) Fluorescence intensity of SOSG in Saos‐2 cells after different treatments. (e) Apoptotic ratio detected by the flow cytometry of Saos‐2 cells exposed to different conditions. (f) CCK‐8 experiment evaluating the biocompatibility of BGS and BGS/I‐LDH scaffolds for hBMSCs up to the seventh day. (g) CLSM images of hBMSCs on the surface of BGS or BGS/I‐LDH stained with 4’,6‐diamidino‐2‐phenylindole (DAPI, blue fluorescence) and rhodamine phalloidin (red fluorescence). (h) Quantitative analysis of the fluorescence intensity of phalloidin in hBMSCs adhered to BGS or BGS/I‐LDH utilizing ImageJ 1.52v software. (i) Digital and optical microscope images indicating the 14‐day alizarin red S staining results of hBMSCs cultured with BGS or BGS/I‐LDH. (j) Quantitative analysis of 14‐day alizarin red S staining images using ImageJ 1.52v software. Statistical comparisons were made by one‐way ANOVA (for multiple comparisons): ^**^ *p* < 0.01, ^***^ *p* < 0.001.

To evaluate the biocompatibility of composite scaffolds, MC3T3‐E1 cells were cultured with BGS and BGS/I‐LDH for 24 h, and the cell viability was assessed by the classical methyl thiazolyl tetrazolium (MTT) method. Compared to the blank group, BGS and BGS/I‐LDH maintained high cell viability, indicating their good biocompatibility (Figure 4a). Subsequently, the co‐culture system of BGS and BGS/I‐LDH was irradiated by a 1270 nm laser (0.75 W cm^−2^, 6 min), where the cell viability of the BGS/I‐LDH group significantly decreased to 9.8% (Figure 4b). We also investigated the photodynamic performance of BGS/I‐LDH on Saos‐2 cells in the presence of various free radical scavengers (superoxide dismutase (SOD, superoxide radical anion scavenger), nicaraven (hydroxyl radical scavenger), catalase (CAT, hydrogen peroxide scavenger), tea polyphenols (TP, ^1^O_2_ scavenger). As shown in Figure S16, after treatment with BGS/I‐LDH+L, the viability of Saos‐2 cells was 9.8%. Expectedly, cell viability of the BGS/I‐LDH+L group were 10.2%, 11.3%, and 9.2% in the presence of SOD, nicaraven, and CAT, respectively, which was significantly increased to 78.6% in the presence of TP, implying that ^1^O_2_ is responsible for the photodynamic activity to induce cell death. To access the killing capability of BGS/I‐LDH against Saos‐2 cells more intuitively, calcein acetoxymethyl ester (Calcein‐AM)/propidium iodide (PI) double staining was conducted. As shown in Figure 4c, the green fluorescence (Calcein) was observed in blank, light, BGS/I‐LDH, and BGS + light groups, while strong red fluorescence (PI) was observed in the BGS/I‐LDH + light group, signifying the complete eradication of tumor cells. Subsequently, SOSG was employed as ^1^O_2_ indicator to evaluate ^1^O_2_ production. As illustrated in Figure 4d and Figure S17, no green fluorescence was detected in the blank, light, BGS/I‐LDH, and BGS + light groups, while a pronounced SOSG fluorescence signal was displayed in the BGS/I‐LDH + light group, indicating the substantial ^1^O_2_ production of BGS/I‐LDH upon 1270 nm laser irradiation. In addition, the cell apoptosis analysis using flow cytometry revealed 90.2% apoptosis of Saos‐2 cells in the BGS/I‐LDH + light group (Figure 4e; Figure S18).

The biocompatibility of BGS/I‐LDH for human bone marrow mesenchymal stem cells (hBMSC) was also evaluated by cell counting kit‐8 (CCK‐8) assays. As shown in Figure 4f, the CCK‐8 optical density (OD) values were comparable across the control, BGS, and BGS/I‐LDH groups on day 1 and days 3. However, higher CCK‐8 OD values were detected in the BGS/I‐LDH group compared with the control and BGS groups on days 5 and 7, indicating that BGS/I‐LDH can gradually promote the proliferation of hBMSCs. To assess the cell‐adhesive properties of BGS/I‐LDH, hBMSCs were directly seeded on the surface of BGS or BGS/I‐LDH and stained with phalloidin for confocal laser scanning microscopy (CLSM) observation. It turned out that hBMSCs adhered and grew uniformly and densely on both BGS and BGS/I‐LDH scaffolds (Figure 4g). Further quantitative analysis revealed a significantly higher fluorescence intensity of phalloidin in the BGS/I‐LDH group than in the BGS group, indicating that the rough surface of BGS/I‐LDH is more beneficial to cell adhesion (Figure 4h). To further investigate the osteogenic differentiation of hBMSCs cultured with different scaffolds, we first analyzed the expression of key osteogenic genes via quantitative polymerase chain reaction (qPCR), including BMP2, RUNX2, and OCN. As shown in Figure S19, both the BGS and BGS/I‐LDH groups significantly upregulated these markers compared to the blank group. Notably, the BGS/I‐LDH group exhibited the highest gene expression levels, indicating a strong capacity to promote osteogenic differentiation at the transcriptional level. To validate these findings at the protein level, we also performed Western blot and corresponding quantitative analysis of BMP2, RUNX2, and OCN. Consistent with the qPCR results, the BGS/I‐LDH group showed the strongest expression of these osteogenic proteins (Figure S20). Additionally, alkaline phosphatase (ALP) activity was assessed on day 7 to evaluate early‐stage osteogenic differentiation. As shown in Figure S21, BGS and BGS/I‐LDH significantly increased ALP activity compared to the blank group, with BGS/I‐LDH demonstrating the highest activity. Furthermore, the in vitro osteogenic properties of BGS/I‐LDH were determined by alizarin red S staining experiments. As shown in Figure 4i (Day 14), compared with the control and BGS groups, more nodular calcium depositions were stained in the BGS/I‐LDH group. Further quantitative analysis indicated a 6.1‐fold and 2.3‐fold increase in OD values of the BGS/I‐LDH group compared with the control and BGS groups, respectively (Figure 4j). By day 21, the nodular calcium depositions in each group were further strengthened, both BGS and BGS/I‐LDH scaffolds significantly enhanced mineralization compared to the control group (Figure S22). Notably, the BGS/I‐LDH group demonstrated a significantly higher level of calcium deposition than the BGS group, indicating that the incorporation of I‐LDH further promotes osteogenic differentiation and late‐stage mineralization of hBMSCs.

### 2.6 In Vivo Evaluation of the Anti‐Osteosarcoma Effect

**FIGURE 5:**
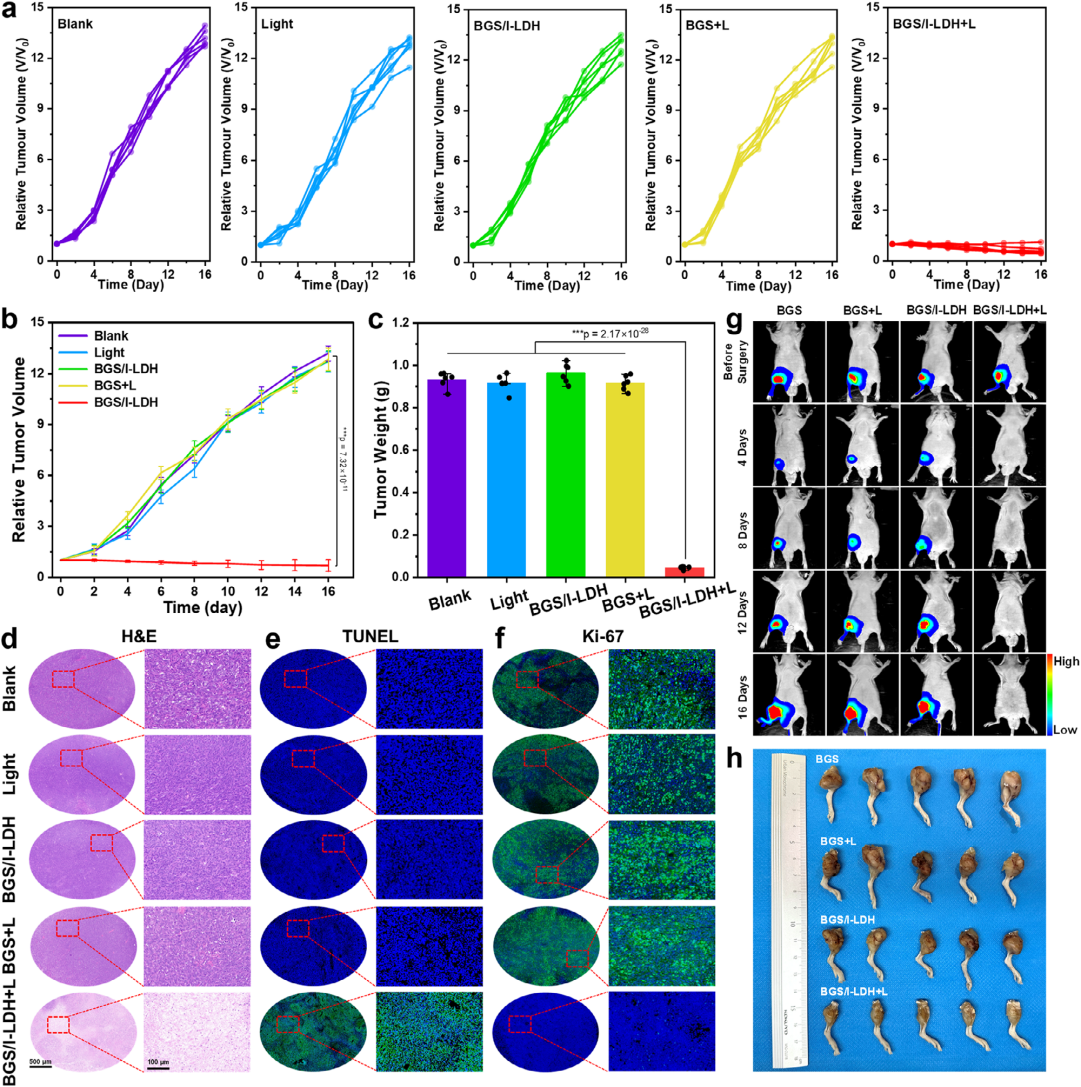
(a,b) Relative tumor growth curves of mice after various treatments: blank, light (0.75 W cm^−2^, 6 min), BGS/I‐LDH, BGS + L, and BGS/I‐LDH + L. Tumor volumes were normalized to initial sizes (*n* = 6). (c) Average weight of Saos‐2 tumors taken on Day 16 in each group. Error bars stand for ± s.d. (*n* = 6). (d) H&E, (e) TUNEL, and (f) Ki‐67 staining of tumor tissue sections from different groups of mice after 16 days of treatment. (g) In vivo bioluminescence imaging of small animals revealing the in‐situ osteosarcoma growth and recurrence before tumor resection surgery, and 4, 8, 12, 16 days after surgery in nude mice treated with indicated therapies. (h) Digital image of in‐situ osteosarcoma specimen in the upper tibia of nude mice treated with indicated therapies, harvested on the 16th day after tumor resection surgery. Statistical comparisons were made by one‐way ANOVA (for multiple comparisons): ^***^ *p* < 0.001.

Encouraged by the excellent anti‐osteosarcoma effect shown in in vitro evaluation, the in vivo PDT effect was evaluated on mice bearing Saos‐2 tumors. Mice were randomly divided into 5 groups: (1) blank, (2) light, (3) BGS/I‐LDH, (4) BGS + light, and (5) BGS/I‐LDH + light. The measurements of tumor volume were carried out every 2 days within 16 days after scaffold implantation. Compared with the blank group, no obvious tumor inhibition was observed in the light, BGS/I‐LDH, and BGS + light groups (Figure 5a,b). Surprisingly, the tumor volume of mice in the BGS/I‐LDH + light group continued to shrink, and the tumor growth was significantly inhibited by 95.2% (Figure S23), demonstrating the excellent in vivo PDT performance of BGS/I‐LDH upon 1270 nm laser irradiation. Similarly, the average tumor weight on Day 16 in the BGS/I‐LDH + light group was only 0.046 g, much lower than that in the blank, light, BGS/I‐LDH, and BGS + light groups (Figure 5c), which was also confirmed by the corresponding gross tumor photos (Figure S24). Additionally, there was no significant change in the body weight of mice across groups during the 16‐day treatment period, indicating negligible toxicity of BGS/I‐LDH (Figure S25). The mice's survival time was also recorded to decide the therapeutic effect of BGS/I‐LDH in vivo. Mice in the blank, light, BGS/I‐LDH, and BGS + light groups all died within 33 days, whereas none of the mice in the BGS/I‐LDH + light group died within 50 days (Figure S26), indicating that the PDT effect of BGS/I‐LDH under 1270 nm laser irradiation significantly improved the survival rate of mice.

The tumor tissue sections were subsequently stained with dihydroethidium (DHE) to detect the ROS level. As shown in Figure S27, strong red fluorescence of DHE was observed only in the BGS/I‐LDH + light group, demonstrating massive ROS generation. Hematoxylin‐eosin (H&E) staining revealed serious damage of tumor tissue in the BGS/I‐LDH + light group (Figure 5d), while the tumor tissue retained its normal morphology in other groups. Similarly, a high level of apoptosis in the BGS/I‐LDH + light group was verified by terminal deoxynucleotidyl transferase (TdT)‐mediated deoxyuridine triphosphate (dUTP) nick end labeling (TUNEL) staining assay (Figure 5e). Moreover, Ki‐67 staining assay was performed, where the BGS/I‐LDH + light group exhibited the weakest green fluorescence (Figure 5f), indicating inhibited cell proliferation in the BGS/I‐LDH group under 1270 nm laser irradiation. Additionally, the in vivo biocompatibility of BGS/I‐LDH was assessed on tumor‐free healthy mice. As shown in Figure S28, no abnormal changes were observed in white blood cell (WBC), red blood cell (RBC), alanine transaminase (ALT), aspartate transaminase (AST), urea (UREA), and other blood routine or biochemical indexes in any group compared with the blank group. H&E staining also revealed no significant pathological abnormalities in major organs of mice across all groups, indicating that BGS/I‐LDH possessed a favorable biosafety, and post‐implantation PDT treatment caused no side effects in mice (Figure S29).

### 2.7 Evaluation of the Anti‐Osteosarcoma Effect and Osteogenic Properties in an In‐Situ Bone Tumor Model

The antitumor efficiency of BGS/I‐LDH for in‐situ bone tumors was also evaluated by establishing tibial osteosarcoma models in nude mice. To better simulate the clinical recurrence of osteosarcoma after resection surgery, tibial osteosarcoma was allowed to grow for 14 days, followed by tumor resection. The bone defects after resection surgery were implanted with BGS or BGS/I‐LDH scaffold and received 1270 nm laser irradiation (0 or 0.75 W cm^−2^) penetrating tibial skin and muscle tissue. In vivo bioluminescence imaging of small animals revealed that the osteosarcoma recurrence was significantly inhibited in the BGS/I‐LDH + light group, while rapid tumor recurrence and expansion were observed in other groups (Figure 5g). Correspondingly, the osteosarcoma‐bearing lower limbs harvested on the 16th day postoperatively presented remarkable differences in the BGS/I‐LDH + light group and other groups, where normal musculature and regular knee joints were observed in the BGS/I‐LDH + light group, while a mass of osteosarcoma tissue invading the knee joint and surrounding soft tissue was found in other groups (Figure 5h).

To simultaneously assess the efficacy of various treatment modalities for both tumor eradication and bone reconstruction in a single animal model, the micro‐CT scanning and reconstruction of the tibiae of osteosarcoma‐resected nude mice implanted with BGS or BGS/I‐LDH, followed with or without PDT, were performed sixteen days post‐implantation (Figure S30). The results indicated significantly extensive bone erosion and deconstruction in the BGS, BGS+L, and BGS/I‐LDH groups, suggesting that further evaluation of osteogenesis and osteointegration in these groups was not warranted. In contrast, the BGS/I‐LDH+L group successfully prevented osteosarcoma recurrence, establishing a prerequisite for the achievement of subsequent favorable scaffold‐bone integration.

### 2.8 In Vivo Evaluation of Osteogenic Properties

**FIGURE 6:**
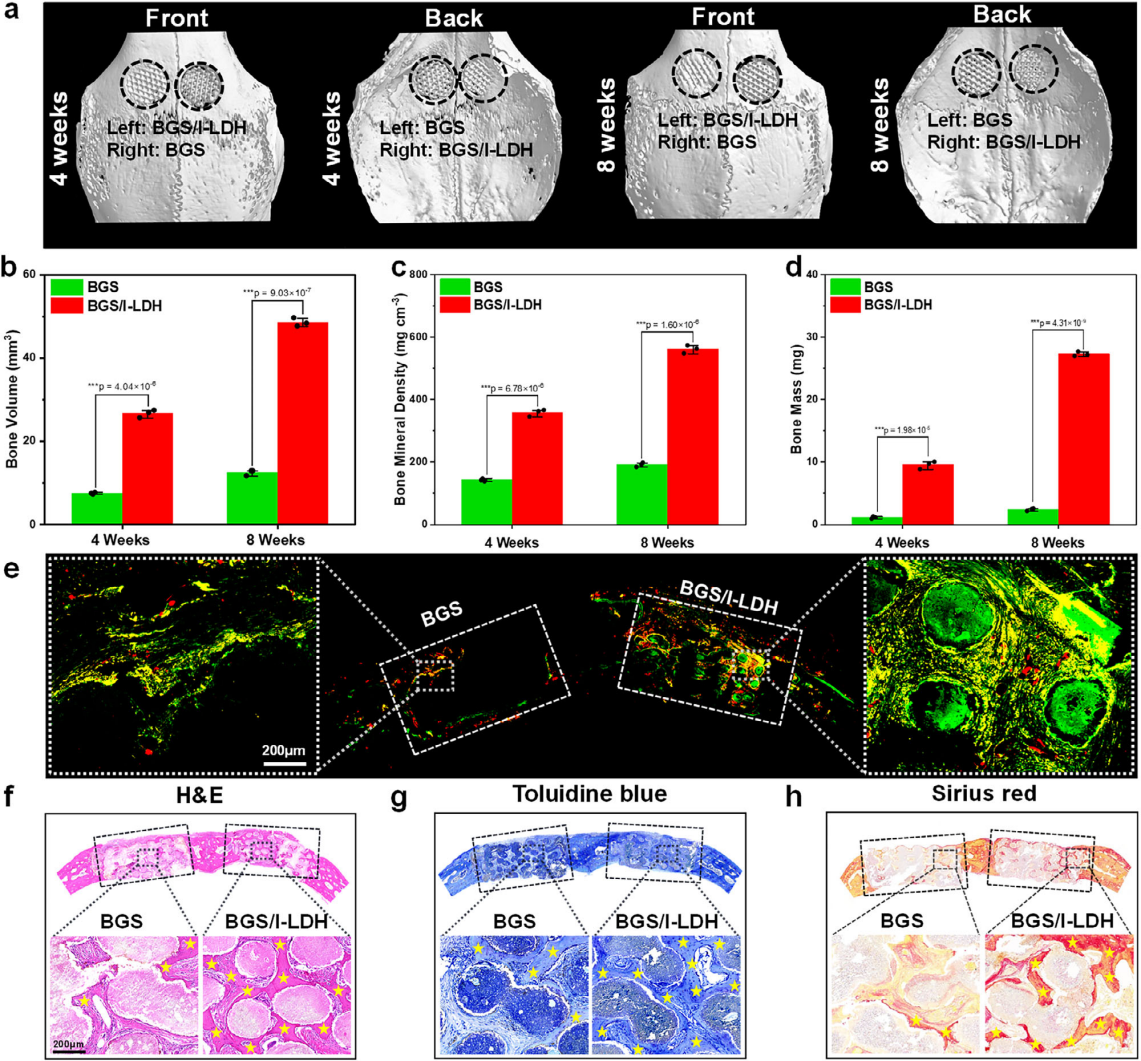
(a) 3D reconstructed Micro‐CT images showing the extent of bone regeneration around BGS and BGS/I‐LDH 4 and 8 weeks after scaffold implantation. (b) Quantitative analysis of the bone volume, (c) bone mineral density, and (d) bone mass with Micro‐CT images. (e) CLSM images highlighting the newly formed bone tissue (labeled with red and green fluorescence). Histological evaluation of the regenerated bone induced by BGS and BGS/I‐LDH using (f) H&E, (g) Toluidine blue, and (h) Sirius red staining. Data are expressed as mean values ± s.d. (*n* = 3). Statistical comparisons were made by one‐way ANOVA (for multiple comparisons): ^***^ *p* < 0.001.

Inspired by the encouraging results of in vitro osteogenic experiments, the in vivo bone regeneration capability of BGS/I‐LDH was investigated in New Zealand white rabbits. Specifically, a two‐hole critical‐sized calvaria defect model was established in rabbits and implanted with BGS and BGS/I‐LDH scaffolds. Four and eight weeks after scaffold implantation, the skulls of rabbits were harvested for Micro‐CT scanning and reconstruction. It turned out that the BGS/I‐LDH induced more remarkable bone regeneration and tight osseointegration than BGS 4 and 8 weeks postoperatively (Figure 6a). Quantitative analysis of Micro‐CT reconstruction images demonstrated 3.4‐fold and 3.8‐fold bone volume, 2.5‐fold and 3.0‐fold bone mineral density, and 8.6‐fold and 11.4‐fold total bone mass increment in the BGS/I‐LDH group compared with the BGS group in weeks 4 and 8, respectively (Figure 6b–d), which is remarkably more preponderant than other previously reported modified BGS (Table S4). In addition, fluorescent Calcein‐AM and alizarin red S were intraperitoneally injected to the calvaria defect rabbits 4 and 6 weeks postoperatively to chelate new calcium deposition and label new bone formation. As presented in Figure 6e, a significant enhancement in fluorescence intensity was detected surrounding the BGS/I‐LDH scaffold, whereas only faint fluorescence was observed around the unmodified BGS, demonstrating the superior capability of BGS/I‐LDH in promoting new bone formation. Subsequently, the sectioned skull samples were stained with H&E (Figure 6f), toluidine blue (Figure 6g), and sirius red (Figure 6h), and significantly regenerated bone integrated with the scaffold was found in the BGS/I‐LDH group, while inconsecutive newly formed bone was seen in the BGS group.

### 2.9 Mechanism of Osteogenic Performances

**FIGURE 7:**
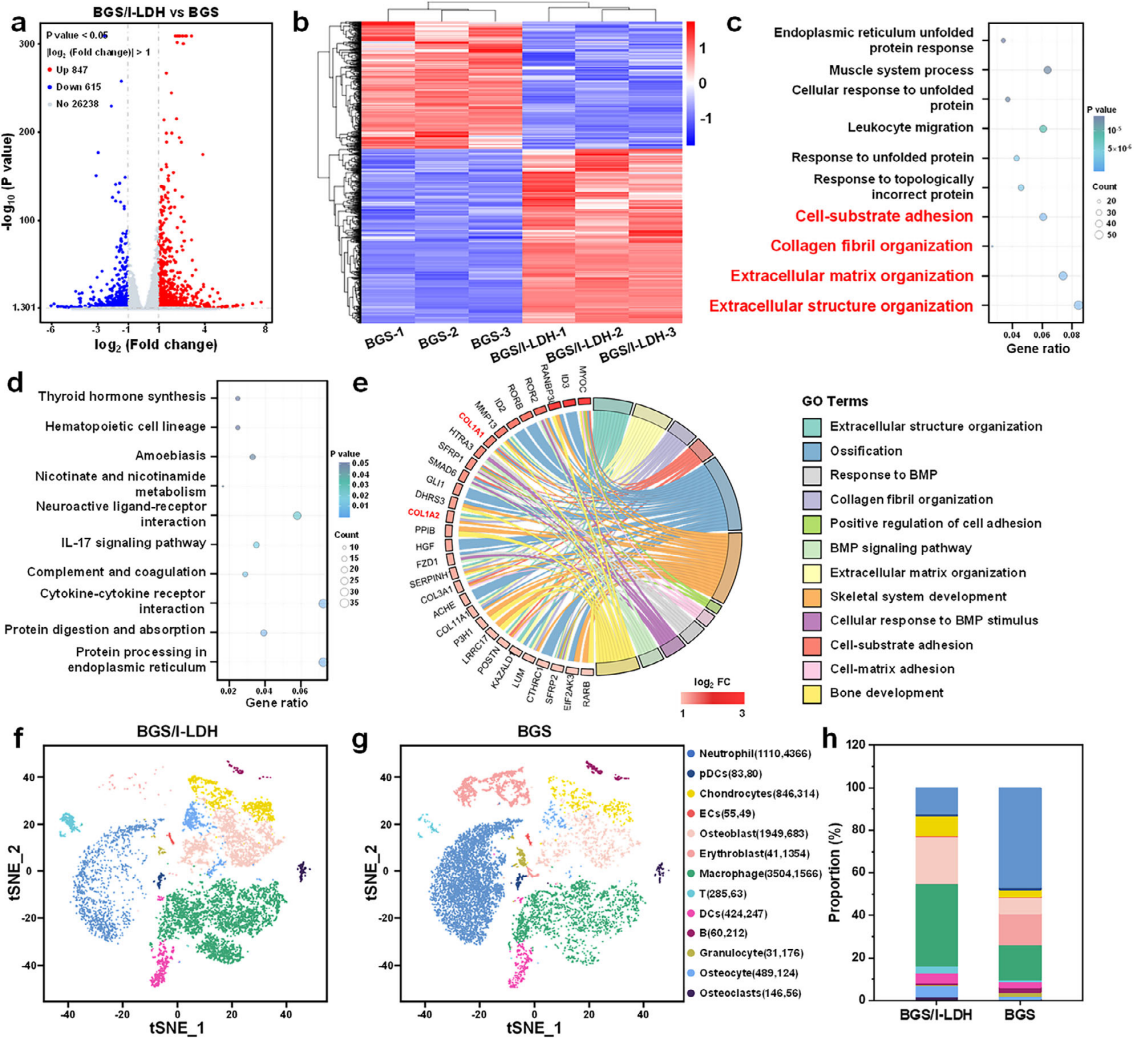
(a) Volcano plot and (b) heatmap of the significantly differentially expressed genes (*p* < 0.05 and |log_2_ FC| > 1) of hBMSCs between the BGS group and the BGS/I‐LDH group determined by transcriptome sequencing. (c) Top 10 enriched biological processes based on the differentially expressed genes identified by GO analysis. The osteogenesis‐related processes were highlighted in red. (d) Top 10 signaling pathways enriched by the differentially expressed genes identified by KEGG analysis. (e) Chord diagram demonstrating the correlation between the top 10 osteogenic biological processes identified in GO analysis and the top 30 differentially expressed genes associated with osteogenesis, ranked by Fold Change values. (f, g) t‐distributed Stochastic Neighbor Embedding (t‐SNE) visualizing cell heterogeneity in reduced dimensions. (h) Semi‐quantitative analysis of the cell clusters, revealing the distribution plots. pDCs, plasmacytoid dendritic cells; ECs, Endothelial cells; DCs, dendritic cells.

Subsequently, the underlying mechanism of the outstanding osteogenic performances of BGS/I‐LDH scaffolds was explored by transcriptome sequencing. As shown in Figure 7a,b, 1462 differentially expressed genes (|log_2_ FoldChange| > 1 & *p* value < 0.05) were identified, among which 615 were down‐regulated (marked in blue), and 847 were up‐regulated (marked in red), suggesting the different cellular states of hBMSCs co‐cultured with BGS/I‐LDH or BGS. These genes were further subjected to Gene Ontology (GO) analysis to explore gene enrichment in various biological processes. Notably, the top 4 enriched biological processes (marked in red) were all closely associated with osteogenesis (Figure 7c), which strongly corroborated the substantial influence of BGS/I‐LDH on the osteogenesis process of hBMSCs. In addition, Kyoto Encyclopedia of Genes and Genomes (KEGG) analysis was performed, and the differentially expressed genes were enriched in the processes associated with cellular cytokine interactions and intracellular protein synthesis and digestion (Figure 7d), indicating that BGS/I‐LDH tended to render the hBMSCs primed for proliferation and differentiation. To further elucidate the specific signaling pathway governing BGS/I‐LDH‐induced osteogenesis, Gene Set Enrichment Analysis (GSEA) was conducted based on the GSEA analysis tool. It was revealed that the Wnt signaling pathway was obviously activated in the BGS/I‐LDH group compared with the BGS group (Figure S31). To visualize the most prominent genes engaged in BGS/I‐LDH‐induced osteogenesis processes, a chord diagram was generated by correlating the top 10 osteogenic biological processes identified in GO analysis with the top 30 differentially expressed genes associated with osteogenesis (ranked by FoldChange) (Figure 7e). Among the identified genes, two noteworthy candidates, COL1A1 and COL1A2 (marked in red), were highlighted as typical osteogenic genes with prominent fold changes. In fact, the COL1A1 and COL1A2 genes collectively encode the synthesis of collagen I, which serves as a fundamental structural component and essential constituent of bone tissue [63]. In addition, the activation of Wnt‐β‐catenin signal pathway could result in the downstream upregulation of COL1A1 and COL1A2, thus promoting osteogenesis. Recent evidence suggests that Mg^2+^ and Zn^2+^ play critical roles in promoting osteogenesis partially through the activation of Wnt/β‐catenin signaling pathway. Mg^2+^ has been shown to promote β‐catenin nuclear translocation and enhance the expression of osteogenic genes such as COL1, partly by elevating intracellular cAMP levels and activating ATF4 (a transcription factor that facilitates Wnt signaling) [64, 65, 66, 67]. In parallel, Zn^2+^ has also been demonstrated to activate Wnt/β‐catenin signaling and stimulate matrix protein synthesis, thereby contributing to bone matrix mineralization and remodeling [68, 69, 70]. Given these findings, the enhanced Wnt/β‐catenin/COL1 signaling pathway was likely attributed to the sustained release of Mg^2+^ and Zn^2+^ from BGS/I‐LDH. However, the identified differentially expressed genes through transcriptome sequencing did not automatically imply corresponding changes in downstream protein expression. Thus, further molecular and morphological verification was needed to validate the engagement of Wnt/β‐catenin/COL1 in BGS/I‐LDH‐induced osteogenesis.

The expression of key genes and proteins involved in the Wnt/β‐catenin/COL1 signaling pathway was further evaluated both in vitro and in vivo. qPCR tests were first conducted to validate the transcriptional levels of Wnt1, β‐catenin, COL1A1, and COL1A2 involved in the Wnt/β‐catenin/COL1 signaling pathway. The qPCR results were consistent with the transcriptome sequencing findings, indicating a significant upregulation in the expression levels of Wnt1, β‐catenin, COL1A1, and COL1A2 in hBMSCs co‐cultured with BGS/I‐LDH compared to the BGS group, with fold changes of 2.8, 3.1, 4.2, and 2.8, respectively (Figure S32). Subsequently, western blot experiments were conducted to assess the protein translation levels of the key genes within the Wnt/β‐catenin/COL1 signaling pathway. As depicted in Figure S33a, a significant increase in the protein expression of Wnt1, β‐catenin, COL1A1, and COL1A2 was detected in the BGS/I‐LDH group compared with the blank and BGS groups. Further quantitative analysis of the protein bands revealed a 2.9‐fold, 3.0‐fold, 2.6‐fold, and 2.8‐fold increment in the fluorescence intensity of Wnt1, β‐catenin, COL1A1, and COL1A2, respectively in the BGS/I‐LDH group compared to the BGS group (Figure S33b), providing robust evidence of the activation of Wnt/β‐catenin/COL1 signaling pathway in response to BGS/I‐LDH treatment. Moreover, in vivo immunohistochemistry and immunofluorescence staining were performed using rabbit skull sections to verify the expression of type I collagen. As shown in Figure S34, significantly intensified immunohistochemical staining and immunofluorescence signal of collagen I intercalating the regenerated bone were observed in the BGS/I‐LDH group compared with the BGS group. Collectively, the in vitro and in vivo investigation and verification provided compelling support for the remarkable osteogenic properties of BGS/I‐LDH mediated by Wnt/β‐catenin/COL1 signaling pathway, which is considered to be attributed to the sustained release of bioactive Mg^2+^ and Zn^2+^ from BGS/I‐LDH.

To investigate differentially regulated cell subsets in response to the BGS/I‐LDH or BGS, single‐cell RNA‐sequencing analysis of the in‐situ cell clusters was performed 2 weeks after scaffold implantation. After filtering, the data were obtained from 18303 cells, with 9280 cells in the BGS group and 9023 cells in the BGS/I‐LDH group. Integrated analysis revealed the presence of 13 distinct subpopulations that emerged during the initial two‐week period of bone repair. As illustrated in Figure 7f,g, a notable increase in osteoblasts (1949 vs. 683) and chondrocytes (846 vs. 314) was found in the BGS/I‐LDH group compared with the BGS group. Furthermore, a significantly greater number of macrophages were identified in the BGS/I‐LDH group compared to the control group (Figure 7h), along with a notably higher proportion of M2‐like macrophages (Figure S35). This shift toward a pro‐regenerative macrophage phenotype indicates that BGS/I‐LDH scaffolds modulate the immune microenvironment to promote bone regeneration rather than sustain chronic inflammation. In summary, single‐cell sequencing provides additional cellular‐level validation of the remarkable osteogenic effects of BGS/I‐LDH.

## 3 Conclusions

In summary, by integrating I‐IPA‐intercalated MgZnAl‐LDH onto BGS, we have developed a novel dual‐functional scaffold, i.e., BGS/I‐LDH, for osteosarcoma recurrence prevention alongside bone reconstruction. As an innovative supramolecular PS, the I‐LDH was constructed through an intercalation strategy and exhibited exceptional photodynamic activity to boost ^1^O_2_ production under 1270 nm laser irradiation, consequently conferring the anti‐osteosarcoma properties of BGS/I‐LDH. Moreover, the BGS/I‐LDH enabled the sustained release of Mg^2+^ and Zn^2+^ ions from MgZnAl‐LDH, facilitating potent and prolonged bone regeneration. In vitro assessments provided corroborating evidence of the heightened photodynamic activity and augmented osteogenic characteristics of BGS/I‐LDH compared with original BGS. In vivo experiments revealed complete osteosarcoma recurrence inhibition and massive new bone formation induced by BGS/I‐LDH, where a 3.8‐fold increment in bone volume, a 3.0‐fold increment in bone mineral density, and an 11.4‐fold increment in new bone mass were noted in BGS/I‐LDH compared to the original BGS after 8 weeks of scaffold implantation, which is remarkably more preponderant than other previously reported modified BGS. Further transcriptome sequencing analysis and molecular verification elucidated the activation of the Wnt/β‐catenin/COL1 signaling pathway, contributing to the osteogenesis processes mediated by BGS/I‐LDH. Single‐cell sequencing further revealed a remarkable increment in osteoblasts, macrophages, and chondrocytes around BGS/I‐LDH compared with pristine BGS. Thus, we have successfully presented a novel dual‐functional alternative for postoperative osteosarcoma management, which exhibited synergetic photodynamic efficiency and osteogenic potentials for osteosarcoma treatment and bone reconstruction.

## Conflicts of Interest

The authors declare no conflicts of interest.

## Supporting information




**Supporting file**: advs74286‐sup‐0001‐SuppMat.docx

## Data Availability

The data that support the findings of this study are available from the corresponding author upon reasonable request.
