# Supplementary material for: A LDH‐Based Supramolecular Photosensitizer‐Functionalized Bioactive Glass Scaffold for Integrated Postoperative Osteosarcoma Recurrence Prevention and Bone Regeneration
Source: Adv Sci (Weinh). 2026 Feb 9;13(22):e24296. doi: 10.1002/advs.202524296 (PMC13088352; doi:10.1002/advs.202524296)
Supplement: Supplementary file 1 — Supporting file: advs74286‐sup‐0001‐SuppMat.docx [file ADVS-13-e24296-s001.docx]

*Supporting Information*

A LDH-based supramolecular photosensitizer-functionalized bioactive glass scaffold for integrated postoperative osteosarcoma recurrence prevention and bone regeneration

Tao Wang, Yixin Bian, Tingting Hu, Mengyang Li, Yuqin Tan, Yu Yang, Xiaolan Jian, Xisheng Weng,* Chaoliang Tan,* and Ruizheng Liang*

T. Wang, M. Li, Y. Yang, and Prof. R. Liang

State Key Laboratory of Chemical Resource Engineering, Beijing Advanced Innovation Center for Soft Matter Science and Engineering, Beijing University of Chemical Technology, Beijing 100029, P. R. China.

E-mail: [liangrz@buct.edu.cn](mailto:liangrz@buct.edu.cn) (R. Liang)

Y. Bian and Prof. X. Weng

Department of Orthopedic Surgery, State Key Laboratory of Complex Severe and Rare Diseases, Peking Union Medical College Hospital, Chinese Academy of Medical Science and Peking Union Medical College, Beijing 100730, P. R. China.

E-mail: [drwengxsh@163.com](mailto:drwengxsh@163.com) (X. Weng)

Dr. T. Hu and Prof. C. Tan

Department of Electrical Engineering & Department of Biomedical Engineering, City University of Hong Kong, Kowloon Tong, Hong Kong SAR 999077, P. R. China.

E-mail: [chaoltan@cityu.edu.hk](mailto:chaoltan@cityu.edu.hk) (C. Tan)

Y. Tan

Graduate School, Hunan University of Chinese Medicine, Changsha, Hunan 410208, China

X. Jian

Hunan Provincial Hospital of Integrated Traditional Chinese and Western Medicine, Changsha, Hunan 410006, China

Prof. C. Tan

Hong Kong Branch of National Precious Metals Material Engineering Research Center (NPMM), City University of Hong Kong, Kowloon Tong, Hong Kong SAR 999077, P. R. China.

Prof. C. Tan

Shenzhen Research Institute, City University of Hong Kong, Shenzhen, 518057, China

Prof. R. Liang

Quzhou Institute for Innovation in Resource Chemical Engineering, Quzhou 324000, P. R. China.

These authors contributed equally: Tao Wang, Yixin Bian and Tingting Hu.

Experimental Section

**Synthesis of MgZnAl-LDH and** **I-LDH nanosheets:** The I-LDH nanosheets were prepared by co-precipitation method. Briefly, 0.25 mmol Mg(NO_3_)_2_·6H_2_O, 0.25 mmol Zn(NO_3_)_2_·6H_2_O and 0.25 mmol Al(NO_3_)_3_·9H_2_O were dissolved in 10 mL DI water as solution A. Solution B (10 mL) contained 1.5 mmol NaOH and 0.5 mmol I-IPA. Then, solution A was gradually added dropwise to solution B under vigorous stirring. 2.4 M NaOH solution was employed to adjust the pH value to 7.0. The whole process was constantly fed with nitrogen flow. Finally, the resultant precipitation was subjected to a hydrothermal Teflon container and processed at 60 °C for 24 h. The resulting I-LDH colloid was washed thoroughly with DI water *via* centrifugation. The preparation of MgZnAl-LDH was the same as above except that I-IPA was not added.

**Synthesis of BGS/I-LDH composite scaffolds:** The BGS were placed in different concentrations (1, 2, 4, 6, 8, 10 mg mL^−1^) of I-LDH suspension for 5 min, followed by ultrasonic treatment for 5 min. Finally, the composite scaffolds were dried thoroughly in an oven at 60 ℃. The whole process was repeated three times.

***In vitro* degradation behavior evaluation**: To assess the *in vitro* degradability of scaffolds, pre-weighed BGS and BGS/I-LDH samples were immersed in phosphate-buffered saline (PBS) solutions at pH 5.4, 6.5 and 7.4. All samples were incubated at 37 °C under static conditions for up to 8 weeks. At 2-week intervals (2, 4, 6, and 8 weeks), the scaffolds were removed, gently rinsed with deionized water, and dried at 60 °C to constant weight. The residual mass was recorded and expressed as a percentage of its initial mass to evaluate the degradation rate at different pH conditions.

***In vivo* degradation behavior evaluation**: To evaluate the *in vivo* degradability of scaffolds, prepared BGS and BGS/I-LDH were sterilized and subcutaneously implanted into the dorsal region of Sprague–Dawley rats. At predetermined time points (2, 4, 6, and 8 weeks post-implantation), rats were euthanized, and the scaffolds were carefully retrieved, rinsed with saline to remove adherent tissues, and dried at 60 °C until a constant weight was achieved. The residual mass of each sample was measured and calculated as a percentage of its initial mass to determine the degradation profile over time.

**Detection of ^1^O_2_ by SOSG:** The generation of ^1^O_2_ was investigated by SOSG assay. Briefly, 200 μL of SOSG (1 mM) and 200 μL of I-LDH (1 mg mL^−1^) were added to the 1600 μL H_2_O, and then exposed to 1270 nm laser irradiation (0.75 W cm^−2^) for 8 min. The fluorescence spectra were recorded by fluorescence spectrometer per minute. The enhanced fluorescence intensity of SOSG at 525 nm indicated the ^1^O_2_ generation. For BGS/I-LDH group, the BGS/I-LDH scaffold was added into the SOSG solution (2 mL, 100 μM).

**Detection of ^1^O_2_ by DPBF:** The generation of ^1^O_2_ was further evaluated by DPBF assay. 200 μL of I-LDH (1 mg mL^−1^) and 30 μL of DPBF (1 mg mL^−1^) were added into 1770 μL H_2_O and mixed thoroughly, followed by 1270 nm laser irradiation (0.75 W cm^−2^) for 8 min. The absorbance of DPBF at 410 nm was monitored per minute. The generation of ^1^O_2_ by BGS/I-LDH was determined *via* similar processes except changing the I-LDH (200 μL, 1 mg mL^−1^) to BGS/I-LDH scaffold.

**Detection of ^1^O_2_ by ESR:** TEMP was used as a trapping agent in ESR measurement to confirm the generation of ^1^O_2_. Specifically, I-LDH solution (100 μL, 100 μg mL^−1^) containing TEMP (45 μM) was exposed to 1270 nm laser irradiation (0.75 W cm^−2^) for 8 min and then examined by ESR.

**Relative ^1^O_2_ quantum yield measurement:** SOSG assay was used to evaluate the ^1^O_2_ quantum yield of I-LDH. 200 μL of SOSG (1 mM) and 200 μL of I-LDH (1 mg mL^−1^) were added to the 1600 μL H_2_O, and then exposed to 1270 nm laser irradiation for 8 min. Free RB was used as a control and irradiated with a 550 nm Xenon lamp for 8 min. The fluorescence spectra were recorded by fluorescence spectrometer per minute. The ^1^O_2_ quantum yield was calculated according to the following formula:

$$\Phi_{\text{I-LDH}}=\Phi_{\text{RB}}\times\frac{A_{\text{RB}}}{A_{\text{I-LDH}}}\times\frac{I_{\text{I-LDH}}}{I_{\text{RB}}}$$

*I*_I-LDH_ and *I*_RB_ are the fluorescence intensity of SOSG at 525 nm after adding RB and I-LDH with 550 nm and 1270 nm laser irradiation, respectively. *A*_I-LDH_ and *A*_RB_ are the absorbance of I-LDH and RB at 1270 nm and 547 nm, respectively. Ф_RB_ is the ^1^O_2_ quantum yield of RB, which is 0.75 in water.

**MTT assay:** Sterilized BGS and BGS/I-LDH scaffolds were used for all subsequent cell and animal experiments. Osteosarcoma Saos-2 cell line was purchased from the Institute of Basic Medical Sciences Chinese Academy of Medical Sciences (Beijing, China), and incubated with McCoy’s 5A (HyClone) medium containing 10% fetal bovine serum and 100 U mL^−1^ penicillin-streptomycin. For cellular cytotoxicity assays, Saos-2 cells (5×10^5^ per well) were seeded on BGS or BGS/I-LDH gently in 48-well plates containing 1 mL medium per well and incubated for 24 h to allow cell adhesion and proliferation onto the scaffold. Afterward, the cells were divided into 5 groups: blank, light (1270 nm, 0.75 W cm^−2^, 6 min), BGS + L, BGS/I-LDH, BGS/I-LDH + L. After incubation at 37 °C for 24 h, the cells on the scaffolds were carefully rinsed with PBS for three times, after which MTT solution (0.5 mg mL^−1^) was added into each well for further 4 h incubation. The cell viability was measured using Microplate Reader.

**Calcein-AM/PI staining:** Calcein-AM/PI staining assay was carried out to confirm the MTT results. Briefly, Saos-2 cells (5×10^5^ per well) were seeded on BGS or BGS/I-LDH in 48-well plates and incubated for 24 h. Subsequently, the cells were treated with or without 1270 nm laser irradiation (0.75 W cm^−2^) for 6 min. After co-staining with Calcein-AM/PI (15 μg mL^−1^), the cells were imaged *via* confocal laser scanning microscopy (CLSM).

**SOSG staining:** SOSG staining assay was performed to confirm the generation of intracellular ^1^O_2_. Saos-2 cells (5×10^5^ per well) were seeded on BGS or BGS/I-LDH in 48-well plates. After incubation for 24 h, the SOSG solution (10 μg mL^−1^) was added into each well for further 0.5 h incubation. Then, the cells were treated with 1270 nm laser irradiation (0.75 W cm^−2^, 6 min). After washing with PBS twice, the fluorescence images of the cells were obtained *via* CLSM.

**Cell apoptosis analysis:** Saos-2 cells were seeded on scaffolds and incubated for 24 h using 48-well plates. Then, the cells were treated with or without 1270 nm laser irradiation (0.75 W cm^−2^) for 6 min. Finally, all treated cells were harvested and stained with Annexin V-FITC/PI assay kit, and then measured by flow cytometry.

**CCK-8 assay:** To access the biocompatibility of BGS and BGS/I-LDH scaffolds, CCK-8 assay was performed by putting BGS or BGS/I-LDH scaffolds in the upper chamber of a 24-well Transwell plate with 3 µm diameter pores while 10^4^ hBMSCs seeding on the lower chamber. The control group put nothing on the upper group and the cell culture medium was replaced daily in all groups. CCK-8 kit was used to detect the proliferation activities of hBMSCs on days 1, 3, 5, and 7 after co-culture. The cell viability was detected by measuring OD values at 450 ± 5 nm in a multifunctional full-wavelength microplate reader.

**Cell adhesion assay:** A total of 10^4^ hBMSCs were spread over BGS or BGS/MZA-I-IPA scaffolds in a 96-well plate and allowed for cell migration and adhesion for 24 h. After that, hBMSCs-adhered scaffolds were immersed in a fluorescent dye solution containing DAPI and rhodamine-phalloidin to obtain corresponding images *via* CLSM.

***In vitro* osteogenic properties evaluation:** BGS or BGS/I-LDH scaffolds were put into the upper chamber of a 24-well Transwell plate with 3 µm diameter pores while 2 × 10^4^ hBMSCs were cultured on the lower chamber. Paraformaldehyde was utilized to fix the cells and Alizarin red S dye was used to stain the calcium nodule deposition produced by osteogenic differentiated hBMSCs after co-culture for 14 and 21 days. Optical inverted microscopy was involved to capture the optical photograph, and a multifunctional full-wavelength microplate reader was used to quantitively detect the Alizarin red S density in a wavelength of 405 nm.

**Alkaline phosphatase (ALP) activity assay:** ALP activity was evaluated using a commercial ALP assay kit (Solarbio, Beijing) following the manufacturer’s instructions. hBMSCs were seeded onto blank wells, BGS, or BGS/I-LDH scaffolds and cultured in osteogenic medium. On day 7, cells were stained with kit and the ALP activity was quantified by measuring absorbance at 405 nm. The results were normalized to the average value of the blank group.

***In vivo* osteosarcoma destruction:** 4-week-old female Balb/c-nude mice obtained from Beijing Vital River Laboratory Animal Technology Co., Ltd were randomly divided into 5 groups (n = 6): (1) blank (control), (2) light, (3) BGS/I-LDH, (4) BGS + L, and (5) BGS/I-LDH + L. Initially, the right hind legs of mice were subcutaneously injected with 100 μL of Saos-2 (10^7^ cells per site). When the volume of tumor reached about 80 mm^3^, the scaffolds (8 mm × 1.5 mm × 1.5 mm) were implanted beneath the tumor under mice anesthesia. One day later, tumors in BGS + L and BGS/I-LDH + L groups were exposed to 1270 nm laser irradiation (0.75 W cm^−2^, 6 min). After that, the tumor volume and body weight of mice were recorded every 2 days up to 16 days. The following formula was used to calculate tumor volume and relative tumor volume.

*Volume = length × width^2^ ×* 0.5

*Relative tumor volume = V_t_/V_0_* (*V_t_*: Tumor volume measured on day *t*. *V_0_*: Tumor volume measured on day 0.)

**Histology examination:** All mice were sacrificed after 16 days of treatment, and tumor tissues were collected for histology examination. Briefly, all tumor tissue sections were stained with Hematoxylin-eosin (H&E), Ki-67 and terminal deoxynucleotidyl transferase (TdT)-mediated deoxyuridine triphosphate (dUTP) nick end labeling (TUNEL) kit, respectively. The final images of stained sections were obtained *via* CLSM.

***In vivo* biosafety:** Fifteen healthy mice were randomly allocated into five groups (n = 3 per group): (1) blank, (2) light, (3) BGS/I-LDH, (4) BGS + L, and (5) BGS/I-LDH + L. Following 16 days of respective treatments, the tail vein blood was collected for blood routine and blood biochemical analysis. Subsequently, all mice were sacrificed for major organ (heart, liver, spleen, lung, kidney) harvesting. The organ sections were subjected to histopathological analysis through H&E staining.

***In vivo* in situ osteosarcoma destruction:** Initially, 1.5 × 10^6^ luciferase labeled-human osteosarcoma cells (143B/LUC) were suspended in 50 μL PBS and subperiosteally injected into the tibia of BALB/c nude mice with 50 μL matrix glue to induce in situ osteosarcoma model. Two weeks after model establishment, the osteosarcomas were surgically excised. A small portion of tumor tissue (approximately 1 mm × 0.3 mm × 0.3 mm) at the edge of the original lesion was intentionally retained during surgical resection to mimic the clinical scenario of incomplete tumor removal. Then, BGS and BGS/I-LDH scaffolds (designed as 1.5 mm × 1.5 mm × 1.5 mm) were implanted into the resected area and received 1270 nm laser irradiation (0.75 W cm^−2^, 6 min) one day after scaffold implantation. *In vivo* imaging of small animals was performed on days 4, 8, 12, and 16 after scaffold implantation using Caliper IVIS Lumina II. Afterward, the osteosarcoma-bearing limbs were harvested and fixed in paraformaldehyde, and then imaged with a digital camera.

***In vivo* osteogenic properties evaluation:** To evaluate the osteogenic capabilities of BGS and BGS/I-LDH scaffolds, critical-sized calvaria defects were made in New Zealand White Rabbits. All surgical experiments were conducted in strict accordance with the National Institutes of Health Guidelines for the use of experimental animals, and all experiments were conducted in accordance with the Guide for the Animal Care and Use Committee of Peking Union Medical College Hospital. Specifically, two parallel 5 mm diameter full-thickness defects were made before the bregma of skull of New Zealand White Rabbits. BGS and BGS/I-LDH were then transplanted to the left defect and right defect, respectively. The critical-sized calvaria defect-modeled rabbits were allowed for 8 weeks for recovery and bone regeneration. During the recovery duration, the fluorescent Calcein-AM and Alizarin red S were intraperitoneally injected at 2, 4, and 6 weeks to label calcium deposition in the defect areas. The skulls were harvested 8 weeks after scaffold transplantation for Micro-CT scanning and 3D reconstruction to access the bone volume, bone mineral density, and bone mass of newly formed bone tissue. Afterward, the skulls were decalcified and sectioned for fluorescence-labeled regenerated bone observation and imaged *via* CLSM. Histopathological sections were further stained with H&E, Toluidine blue, and Sirius red to determine the osteogenesis and osteointegration between the scaffolds and the host bone tissue.

**Transcriptome sequencing:** One hundred thousand hBMSCs were cultured on the lower chamber of a 6-well Transwell plate with 3 µm diameter pores for 7 days with the BGS and BGS/I-LDH placed in the upper chamber. The total RNA of co-cultured hBMSCs was extracted by TRIzol and an RNA extraction kit. Afterward, magnetic beads with Oligo (dT) were utilized to enrich the extracted RNA, which were subsequently interrupted by interrupting reagents. The one-strand cDNA was synthesized based on the interrupted mRNA and six-base random primers while the double-strand cDNA was synthesized with a double-strand reaction system. Agilent 2100 Bioanalyzer sequencer was involved to access the constructed library, and the Illumina sequencer was used for transcriptome sequencing. R Foundation for Statistical Computing was involved in data analyses and image production.

**qRT-PCR analysis:** The protocol of RNA extraction was the same as that in the section of *Transcriptome sequencing*. The concentration of extracted RNA was determined by a NanoDrop spectrophotometer. Afterward, an RNA-to-cDNA kit was used to transcribe RNA to cDNA. The transcribed cDNA was then enriched by a SYBR Green RT-PCR kit in ABI Step One Plus real-time PCR system. ∆∆cT method was used to detect the relative expression level of target genes, for which GAPDH served as the reference gene.

**Western-blot assay:** The cell-scaffold co-culture paradigm was the same as that in *Transcriptome sequencing* section. The total protein extraction and quantification followed the manufacturer’s instruction of RIPA Lysis and Extraction Kit and Pierce TM Rapid Gold BCA Protein Assay Kit. Subsequently, extracted proteins were transferred to a polyvinylidene difluoride (PVDF) membrane, which was incubated with primary antibodies at 4 °C for 12 h and second antibodies at room temperature for half an hour. The target protein images were visualized by Enhanced Chemiluminescence and analyzed by Bio-Rad image analysis software.

**Immunohistochemistry and immunofluorescence:** The sample of rabbit skull sections was obtained following the protocol in the section of *In vivo osteogenic properties evaluation*, which was further digested and blocked by Proteinase K and 3% H_2_O_2_. The primary antibody and second antibody were sequentially incubated with skull sections at 4 °C and 25 °C, respectively. Optical inverted microscopy and CLSM microscope were utilized to capture the immunohistochemistry and immunofluorescence images.

**Single-cell RNA-sequencing analysis:** Two weeks after implantation of either BGS or BGS/I-LDH, three rabbits were euthanized for each group. Tissue regenerated around the scaffold was carefully dissected, minced, and rinsed repeatedly with 4°C PBS. Subsequently, the tissue was enzymatically digested to isolate individual cells. mRNA libraries were then prepared and subjected to sequencing. To ensure data quality, cells with over 300 detectable genes, a minimum of 500 read counts, and less than 20% mitochondrial gene expression were retained. Following the Seurat pipeline, the data was log-normalized and scaled. t-distributed Stochastic Neighbor Embedding (t-SNE) were used to visualize cellular diversity in reduced dimensions.

**Statistical analysis:** Data are expressed as mean ± standard deviation (S.D). Statistical comparisons were made by one-way ANOVA: *p < 0.05, **p < 0.01, ***p < 0.001.


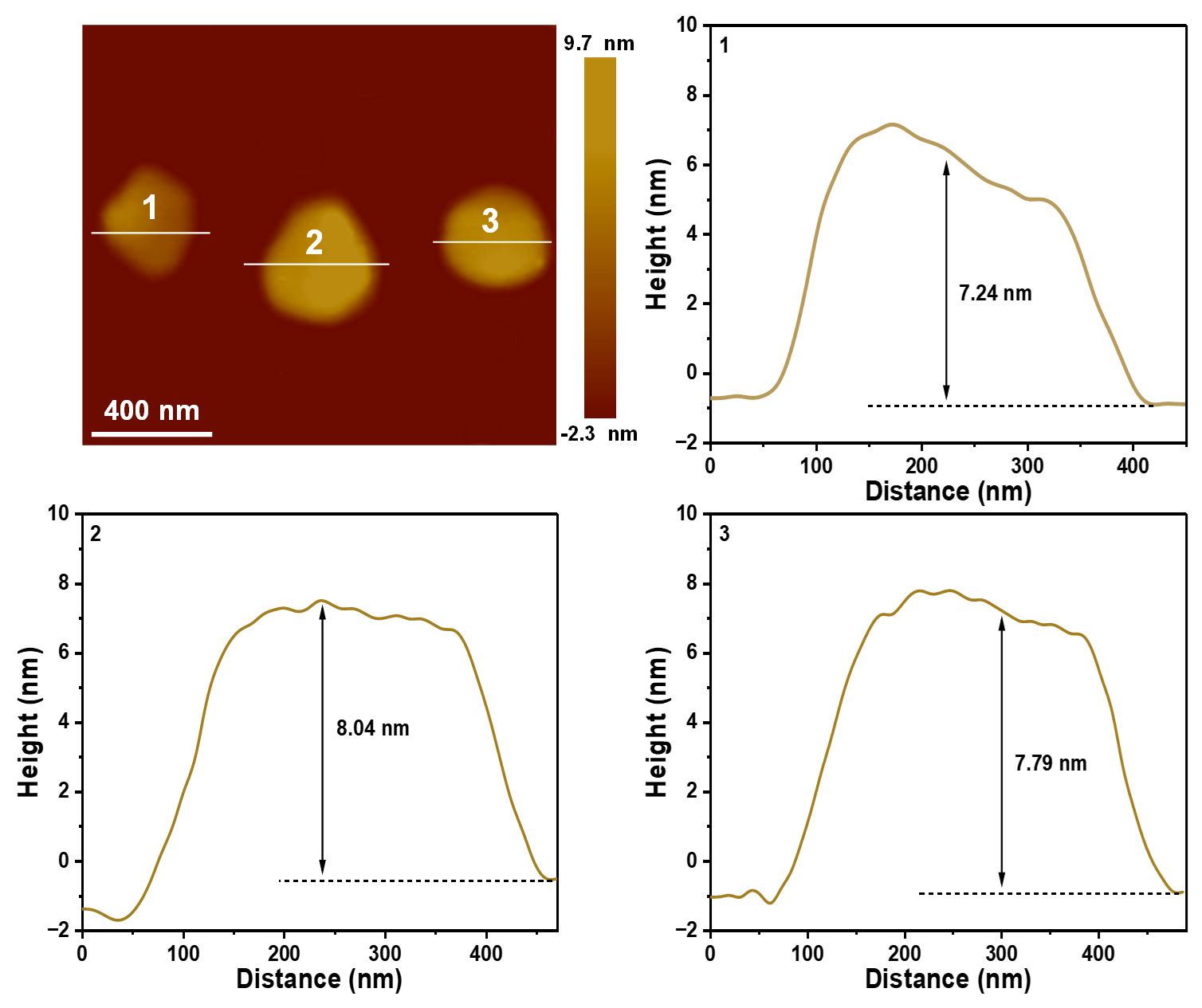


**Figure S1.** The AFM image of I-LDH nanosheets and corresponding height analysis.

**Figure S2.** The XRD pattern of MgZnAl-LDH.


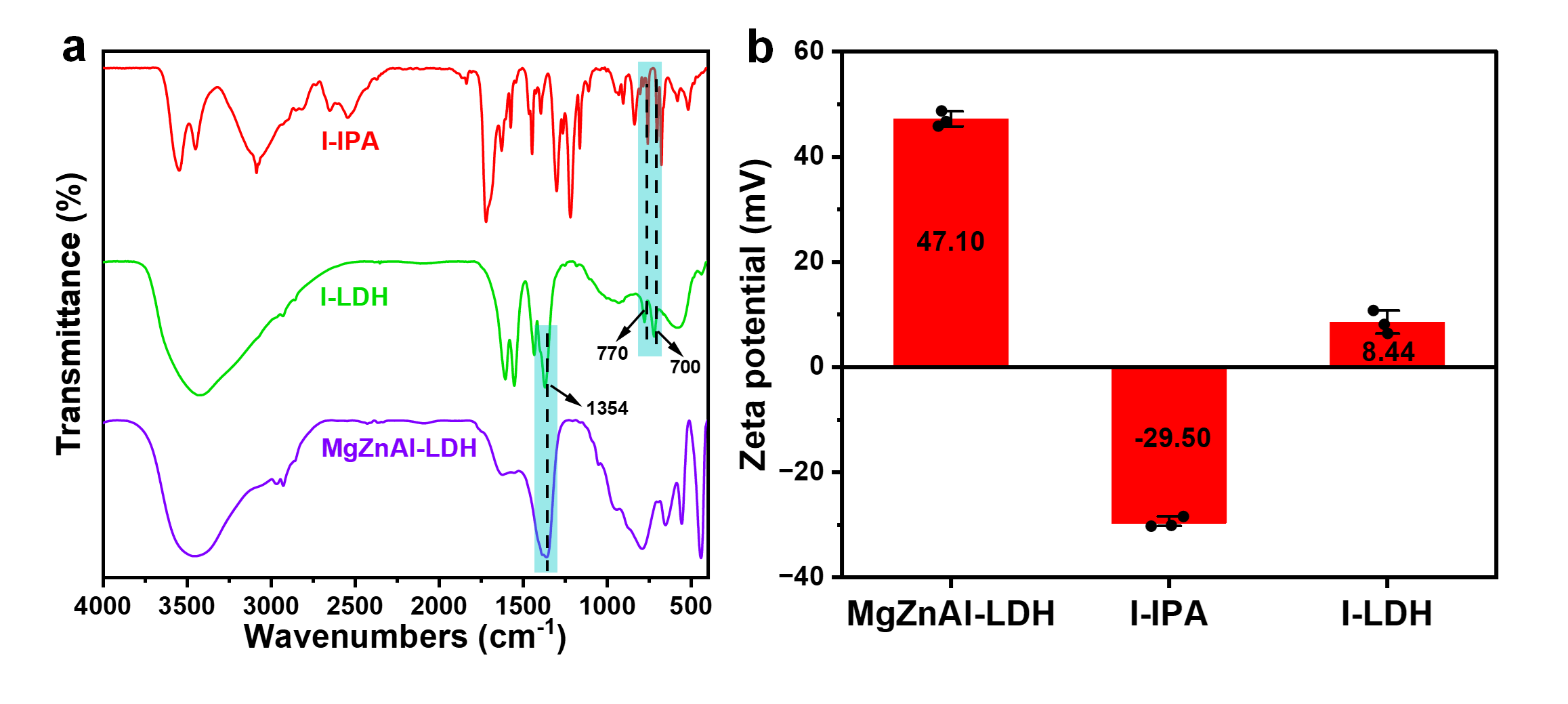


**Figure S3.** a) FT-IR spectra and b) Zeta potential analysis of I-IPA, MgZnAl-LDH and I-LDH.


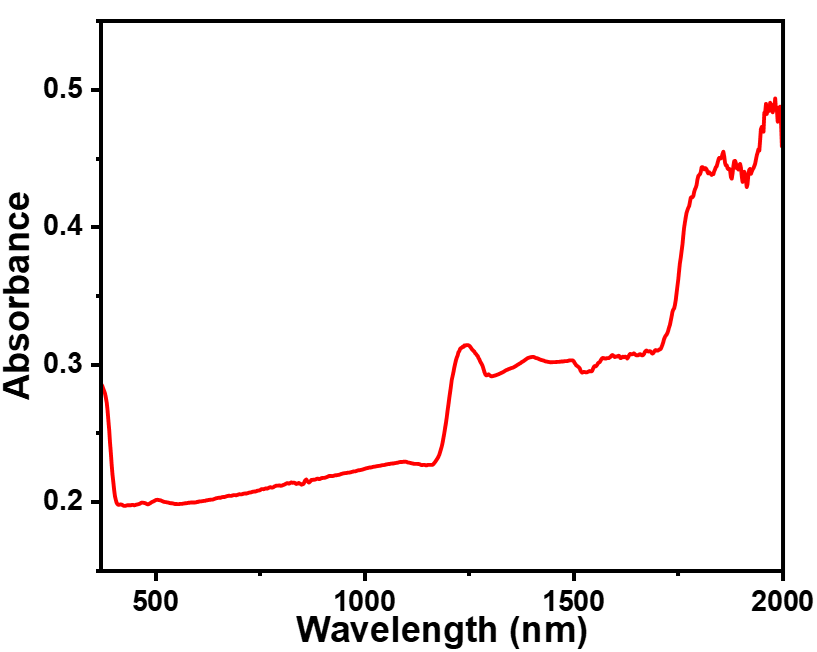


**Figure S4.** UV-vis-NIR absorption spectrum of I-LDH.


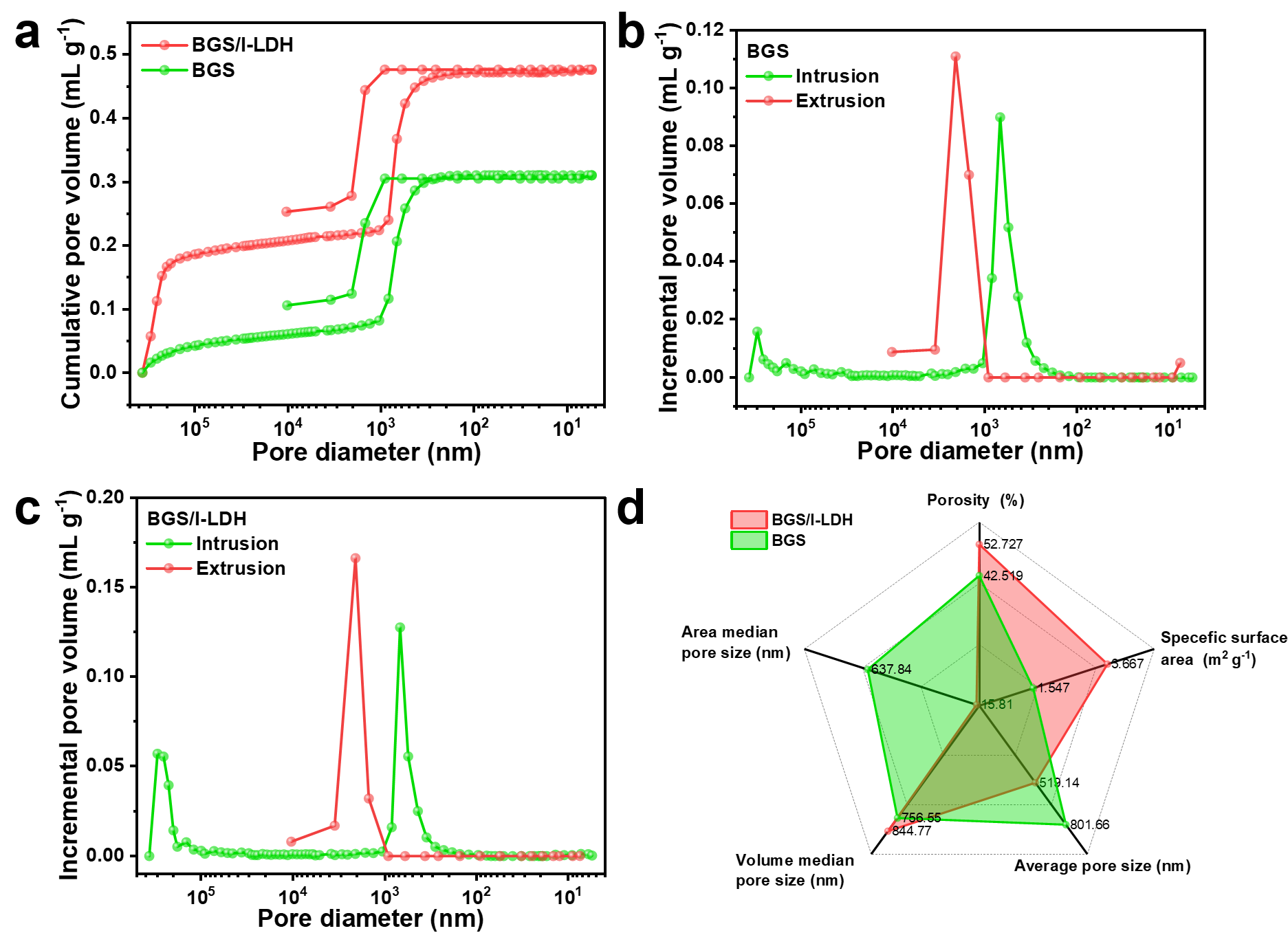


**Figure S5.** Mercury Intrusion Porosimetry (MIP) test results for BGS and BGS/I-LDH.

**Figure S6.** a) Stress-strain curve, b) compression modulus and strength of scaffolds before and after I-LDH deposition.

**
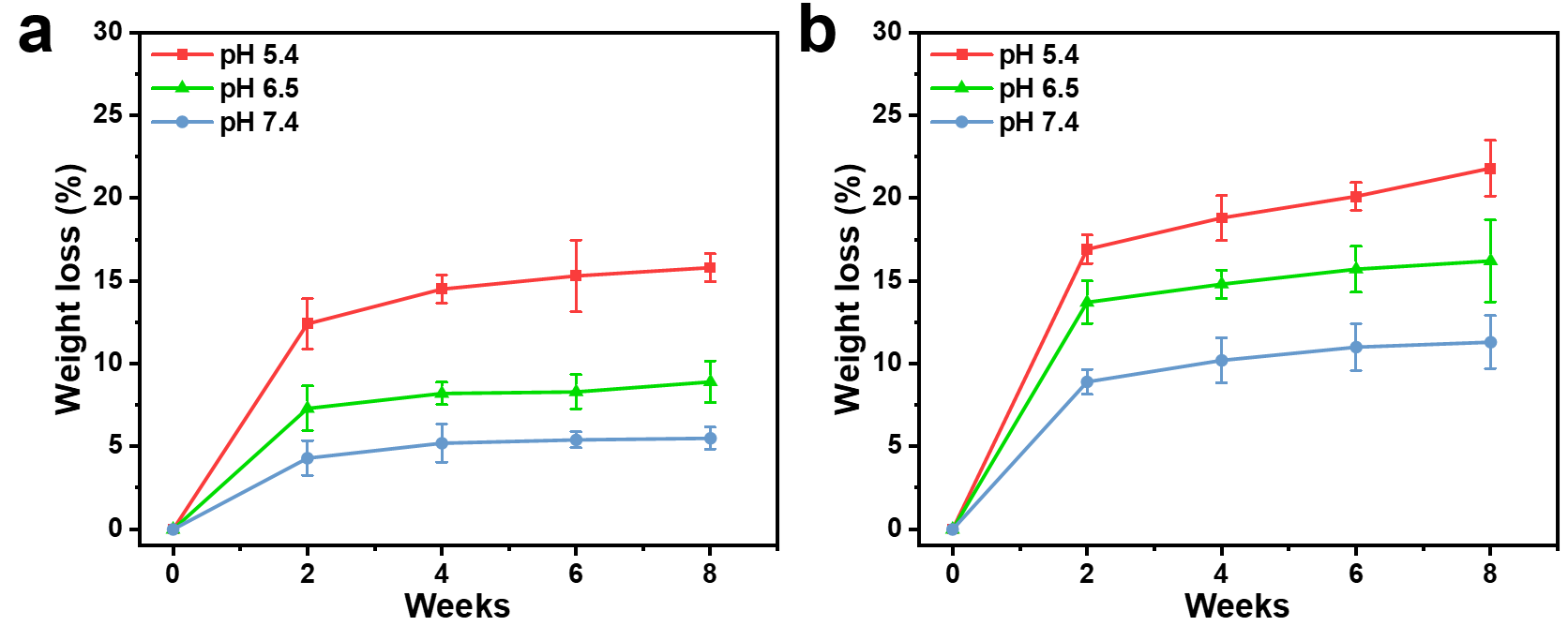
**

**Figure S7.** *In vitro* degradation behaviours of a) BGS and b) BGS/I-LDH in PBS solutions at pH 5.4, 6.5, and 7.4.

**
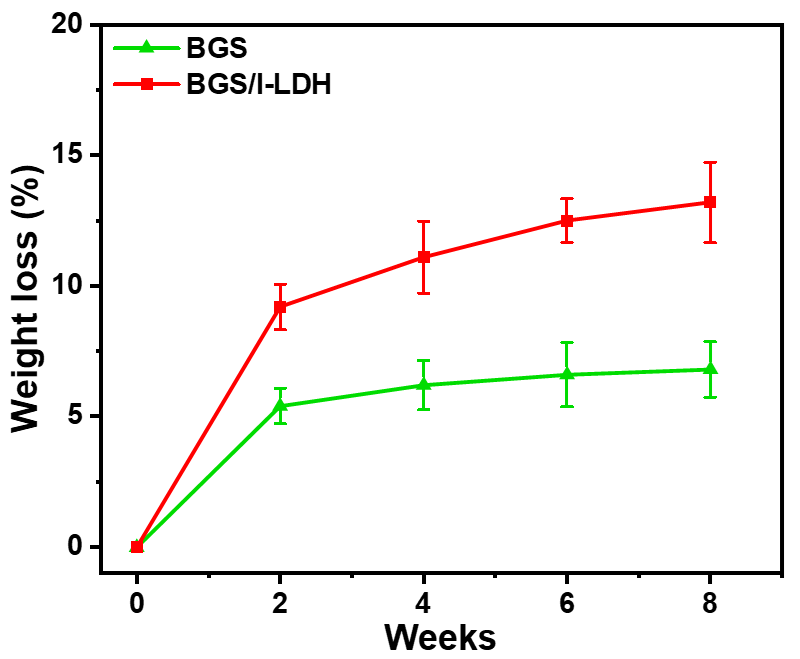
**

**Figure S8.** *In vivo* degradation behaviour of BGS and BGS/I-LDH over 8 weeks after subcutaneous implantation in rats.


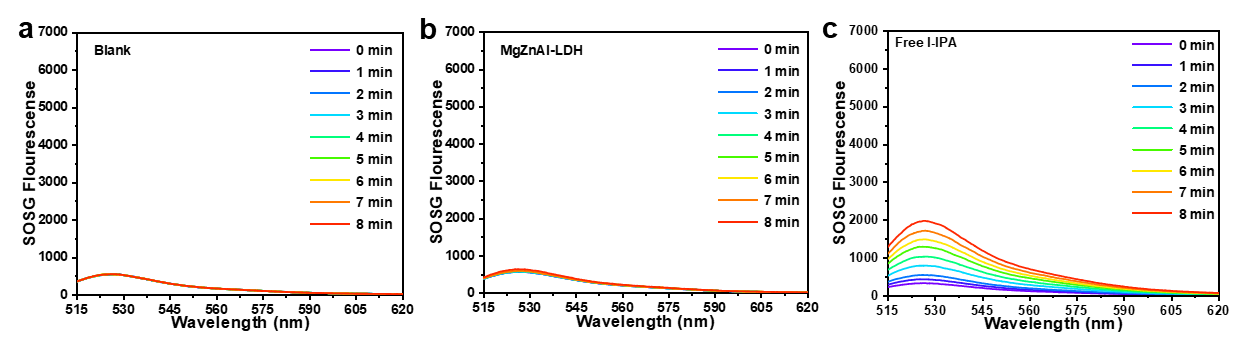


**Figure S9.** The fluorescence spectra of a) SOSG in blank, b) MgZnAl-LDH, and (**c**) free I-IPA under 1270 nm laser irradiation (0.75 W cm^−2^).

**Figure S10.** The UV-vis spectra of a) DPBF in blank, b) MgZnAl-LDH, and c) free I-IPA under 1270 nm laser irradiation (0.75 W cm^−2^).

**Figure S11.** a) The time-variation ESR signal intensity of TEMP/^1^O_2_ in the presence of I-LDH under 1270 nm laser irradiation (0.75 W cm^−2^) and b) the magnified area marked by the dash line rectangle in a).

**Figure S12.** a) The UV-vis spectra of Rose Bengal (green) and I-LDH (red). The fluorescence spectra of SOSG in the presence of b) Rose Bengal under 550 nm Xenon lamp irradiation (0.75 W cm^−2^) and c) I-LDH under 1270 nm laser irradiation (0.75 W cm^−2^).

**Figure S13.** The fluorescence spectra of SOSG in the presence of BGS/I-LDH scaffolds at different impregnation concentrations (1, 2, 3, 4, 6, 8, 10 mg mL^−1^) under 1270 nm laser irradiation (0.75 W cm^−2^).

 **Figure S14.** The UV-vis spectra of DPBF in the presence of BGS/I-LDH scaffolds at different impregnation concentrations (1, 2, 4, 6, 8, 10 mg mL^−1^) under 1270 nm laser irradiation (0.75 W cm^−2^).


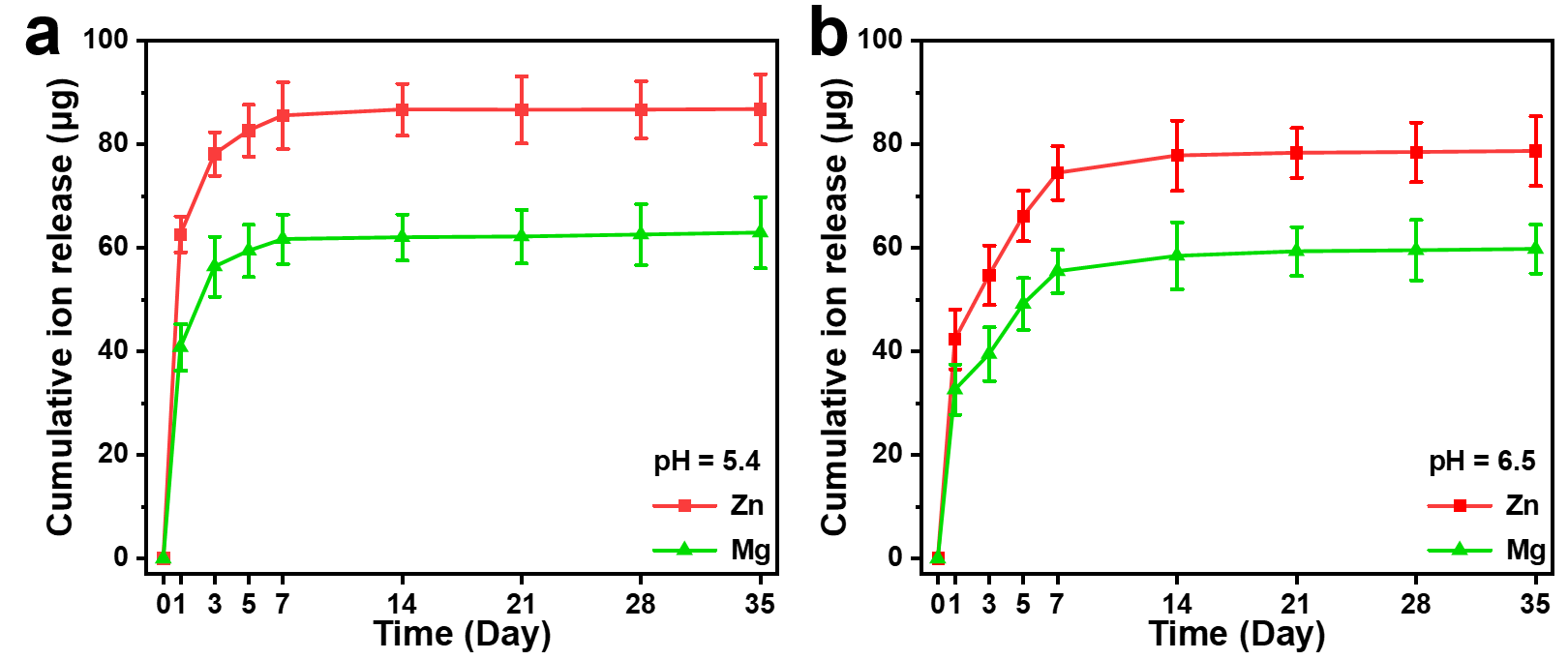


**Figure S15.** Zn and Mg ions release kinetics of the BGS/I-LDH under simulated environments at pH a) 5.4 and b) 6.5.


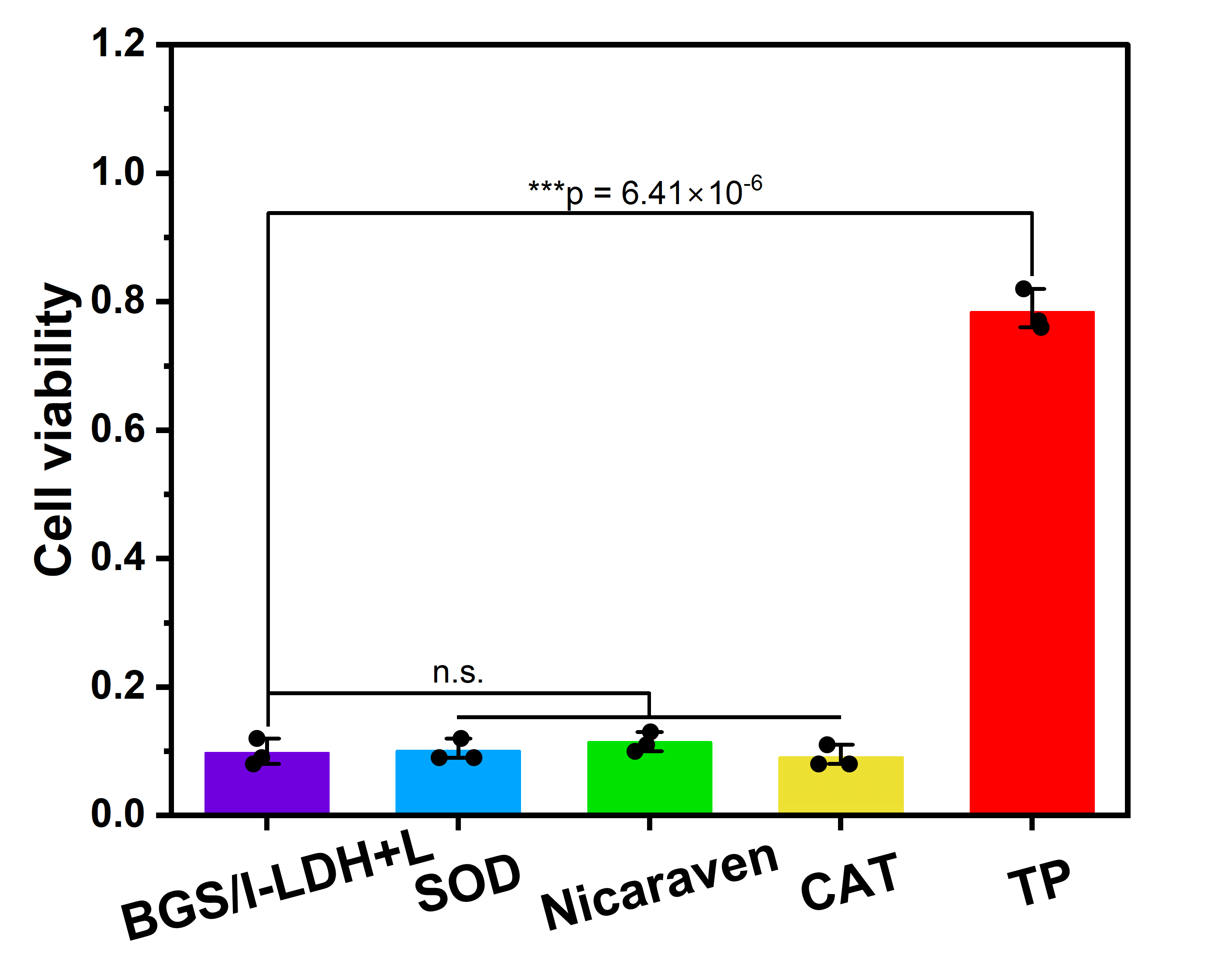


**Figure S16.** Cell viability of Saos-2 cells treated with BGS/I-LDH+L in the presence of different free radical scavengers: SOD, nicaraven, CAT, TP.


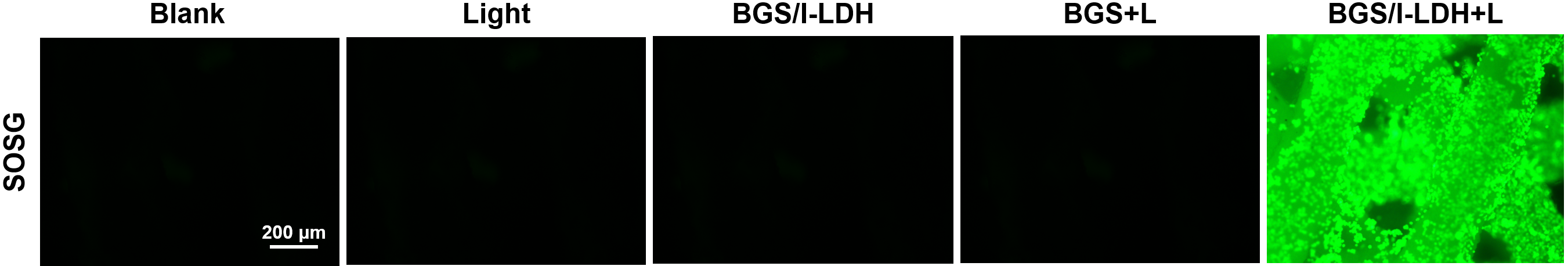


**Figure S17.** SOSG staining images of Saos-2 cells seeded on the BGS and BGS/I-LDH after different treatments.


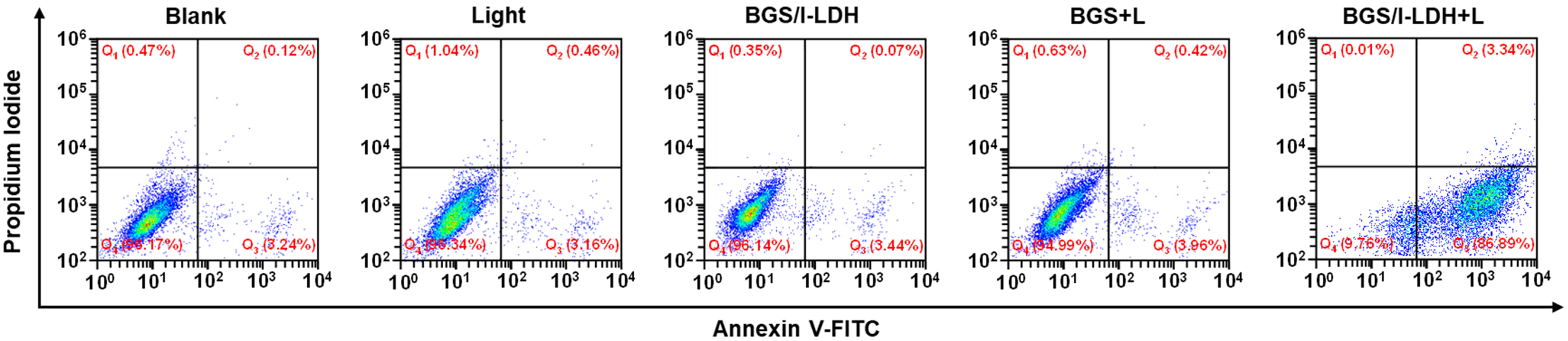


**Figure S18.** Cell apoptosis analysis using Annexin V-FITC/PI double staining method.


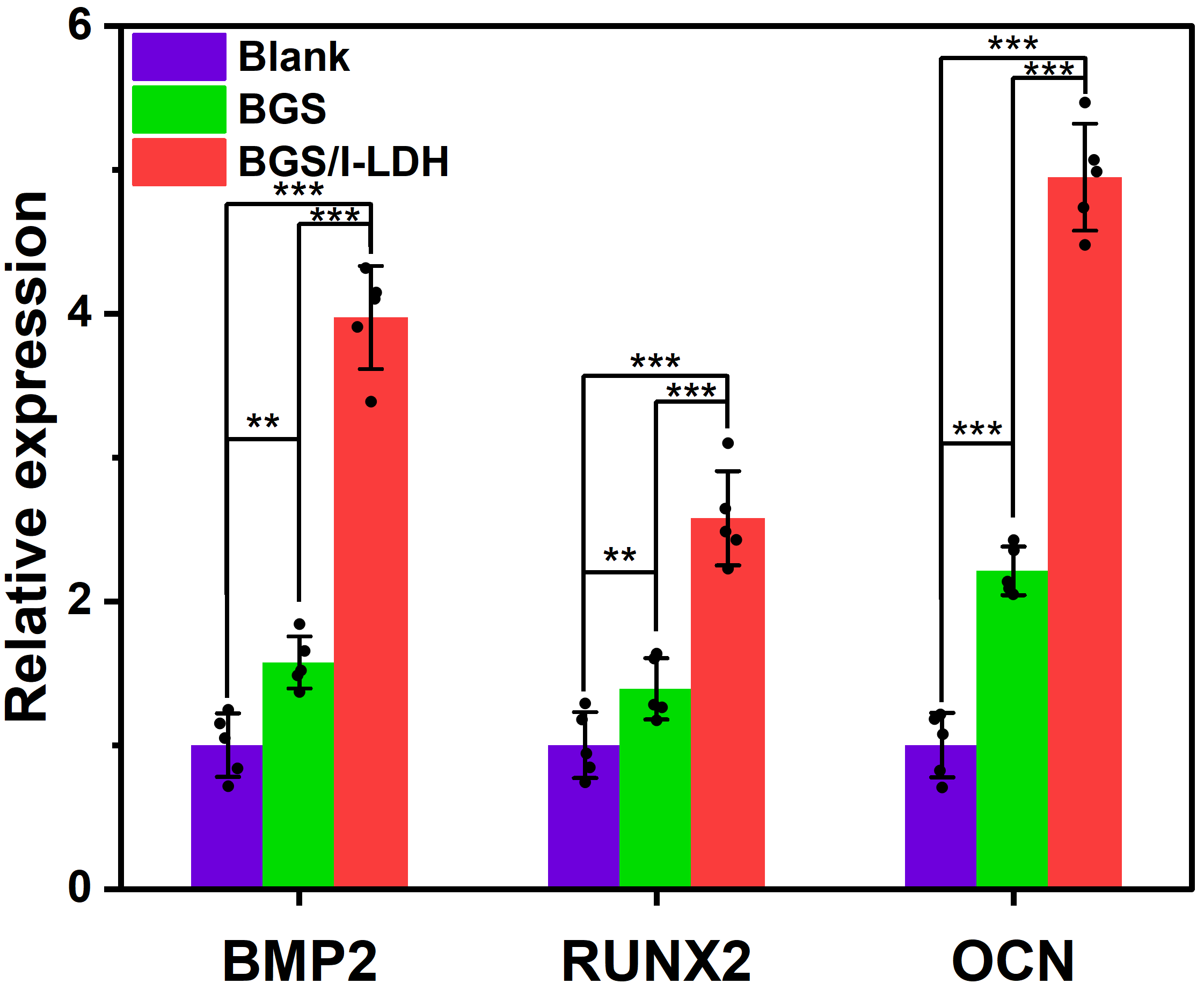


**Figure S19.** Relative mRNA expression levels of osteogenic markers (BMP2, RUNX2, and OCN) in hBMSCs cultured with different scaffolds. Statistical comparisons were made by one-way ANOVA (for multiple comparisons): **p < 0.01, ***p < 0.001.


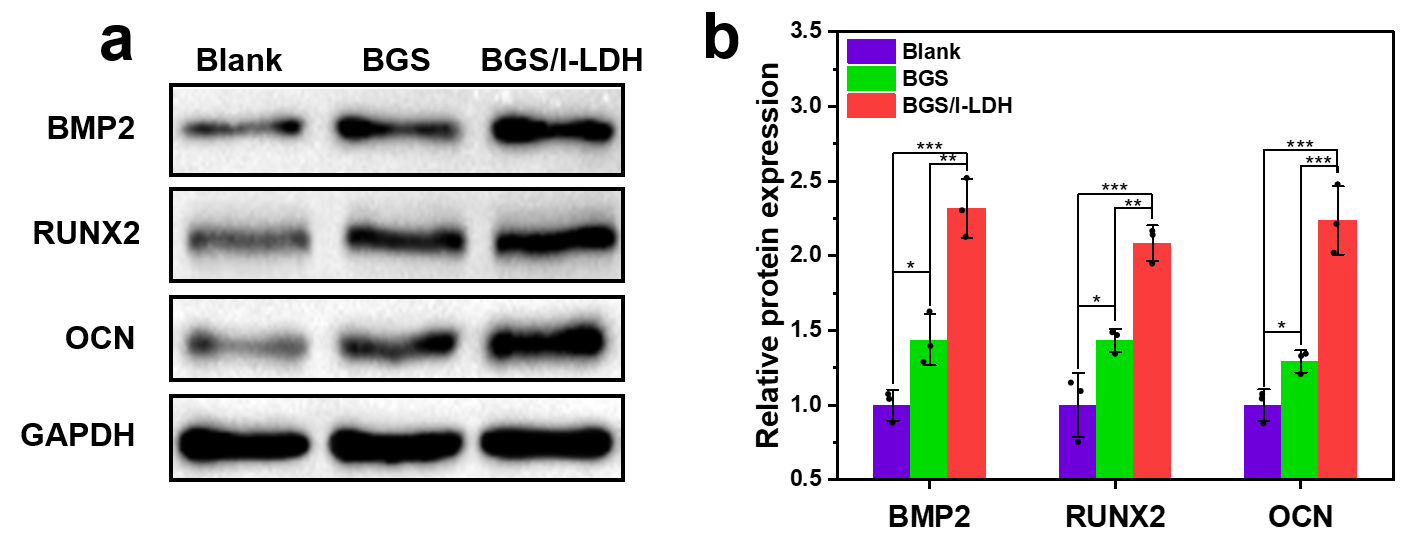


**Figure S20.** Western blot and quantitative analysis of BMP2, RUNX2, and OCN protein expression in hBMSCs after 7 days of culture on different scaffolds. Statistical comparisons were made by one-way ANOVA (for multiple comparisons): *p < 0.05, **p < 0.01, ***p < 0.001.


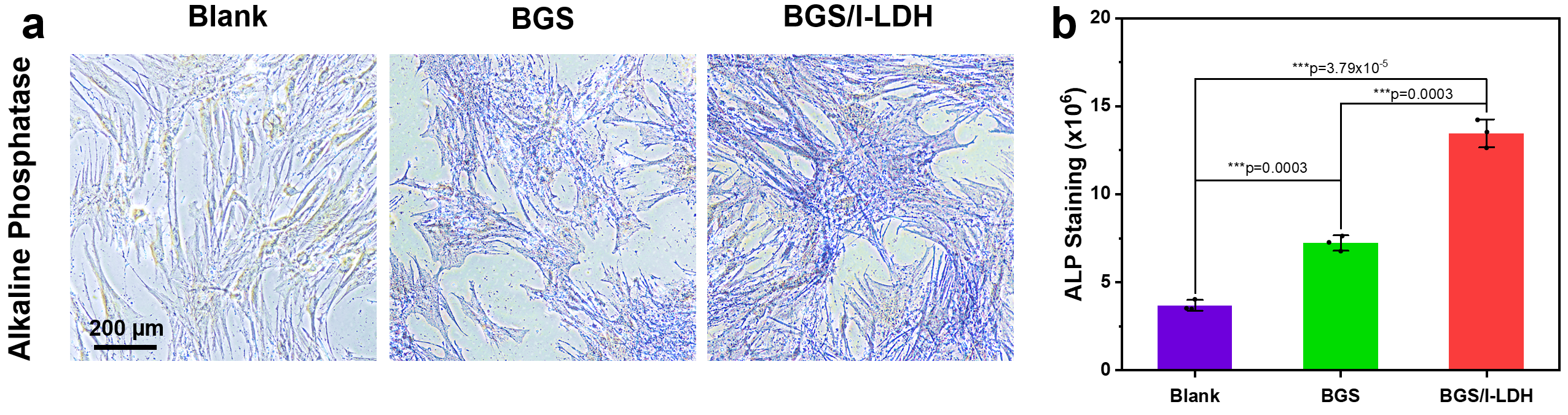


**Figure S21.** a) Optical microscope images indicating the ALP results of hBMSCs cultured with BGS or BGS/I-LDH. b) Quantitative analysis of ALP staining images using ImageJ 1.52v software. Statistical comparisons were made by one-way ANOVA (for multiple comparisons): ***p < 0.001.


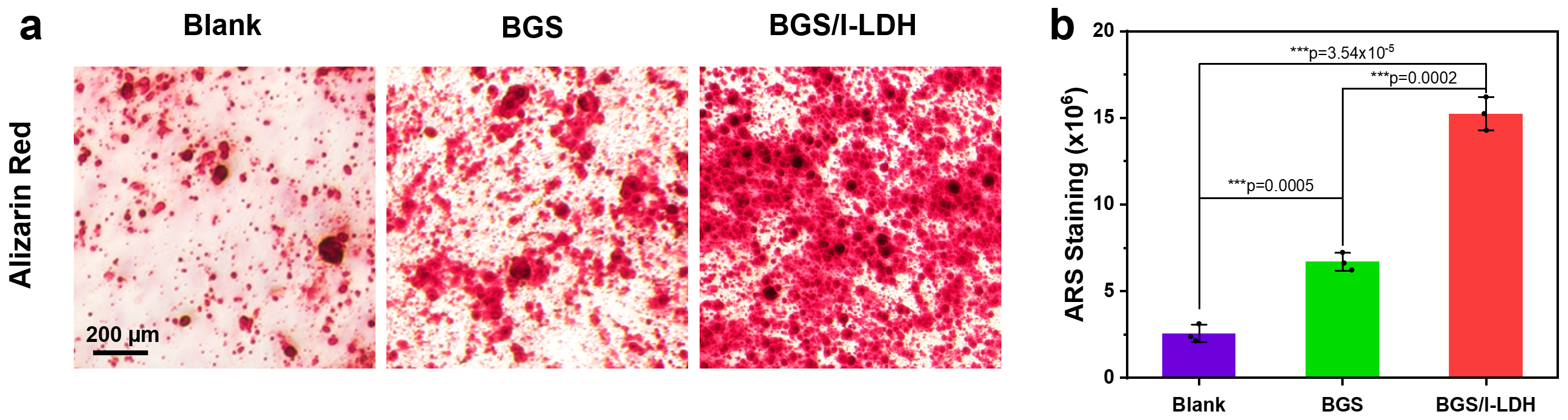


**Figure S22.** a) Optical microscope images indicating the 21-day alizarin red S staining results of hBMSCs cultured with BGS or BGS/I-LDH. b) Quantitative analysis of 21-day alizarin red S staining images using ImageJ 1.52v software. Statistical comparisons were made by one-way ANOVA (for multiple comparisons): ***p < 0.001.

**Figure S23.** The relative tumor growth inhibition rate of each group measured on the 16th day.


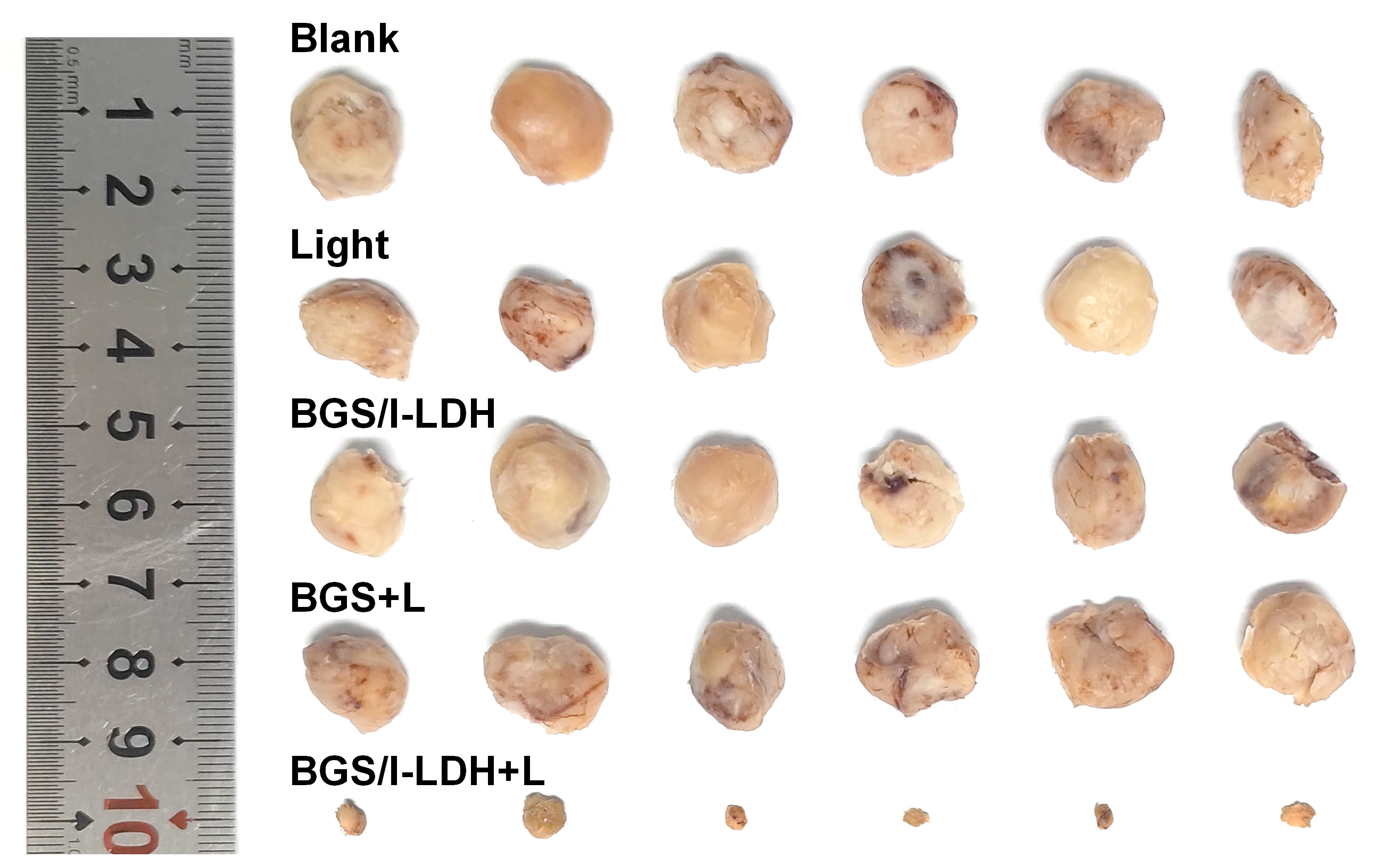


**Figure S24.** Corresponding digital photos of Saos-2 tumor taken on Day 16 in each group.

**Figure S25.** Bodyweight changes of Saos-2 tumor-bearing mice under different treatments for 16 days.

**Figure S26**. Survival rates of Saos-2 tumor-bearing mice.


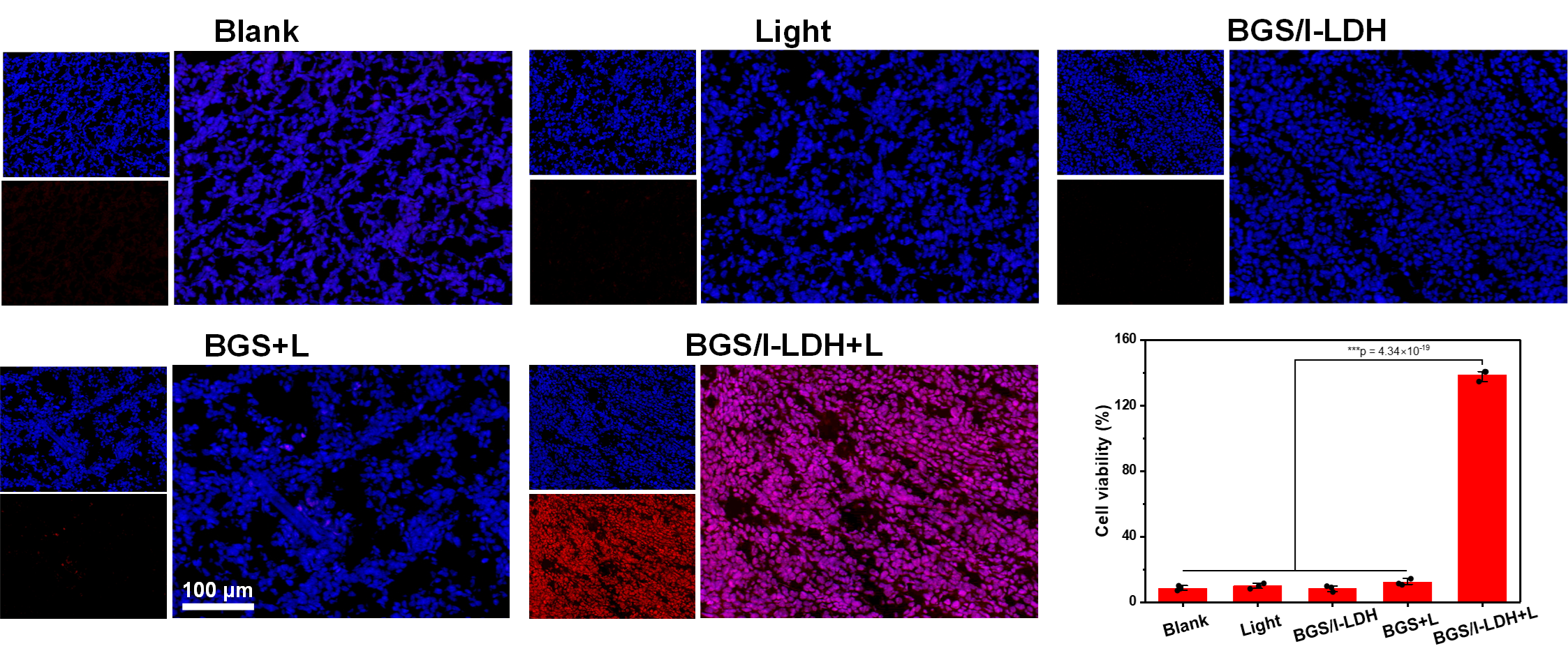


**Figure S27**. The DHE staining images of tumor tissue sections and quantitative analysis of its fluorescence intensity.


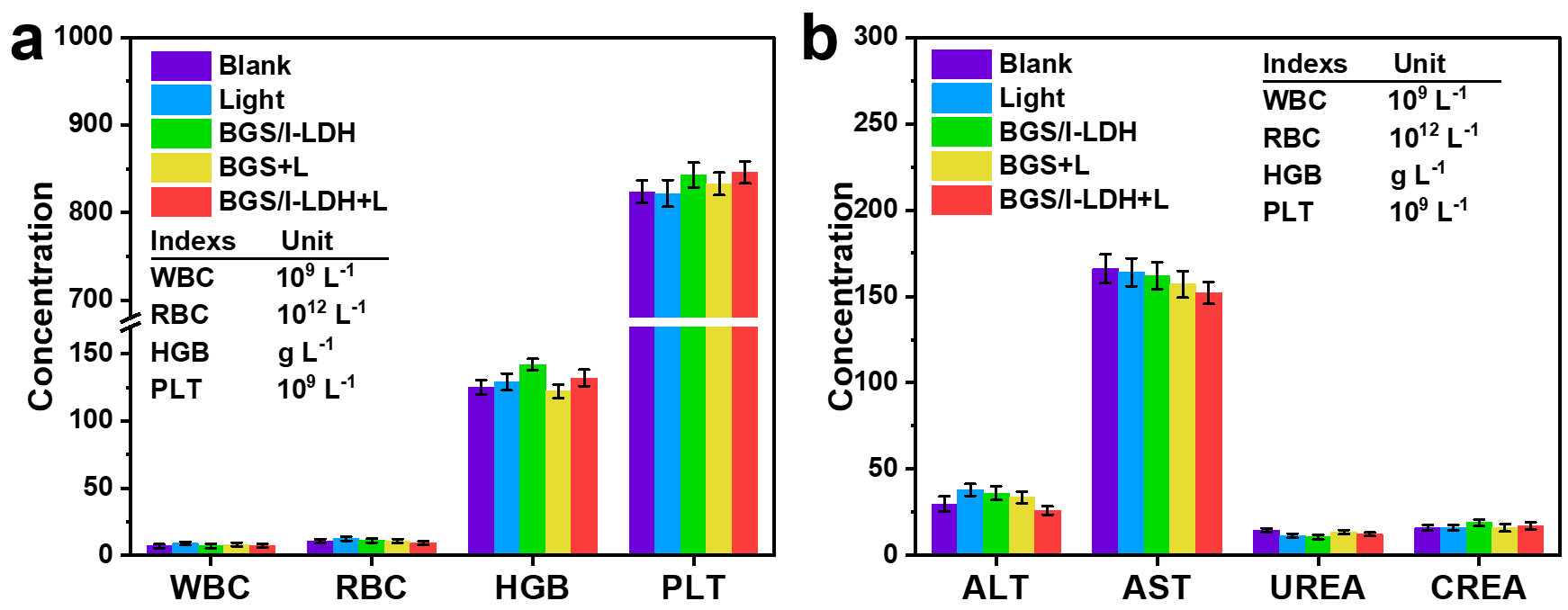


**Figure S28**. Blood routine and blood biochemical indexes of mice on the 16th day after the implantation of scaffolds.


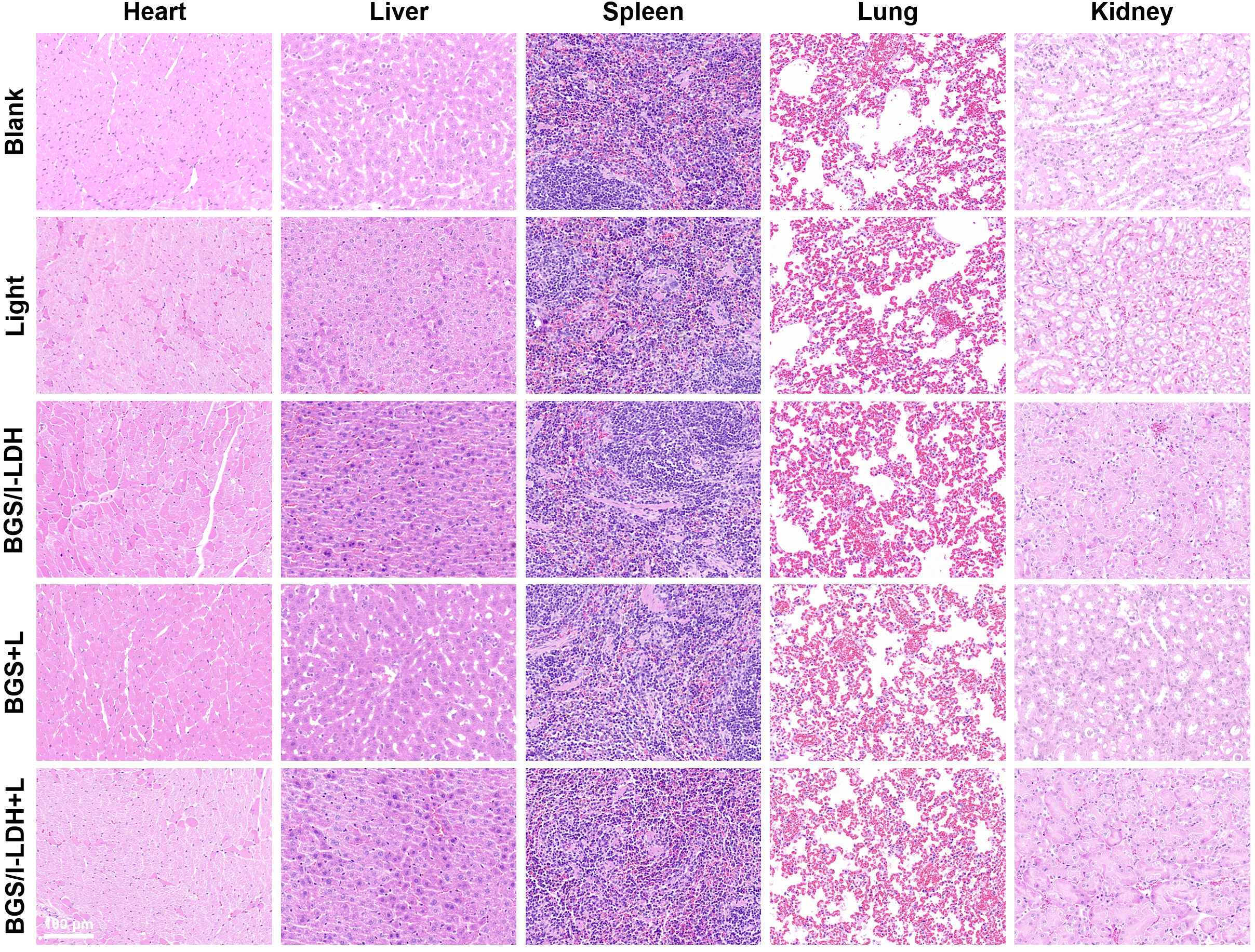


**Figure S29.** H&E staining images of major organs (heart, liver, spleen, lung, kidney).


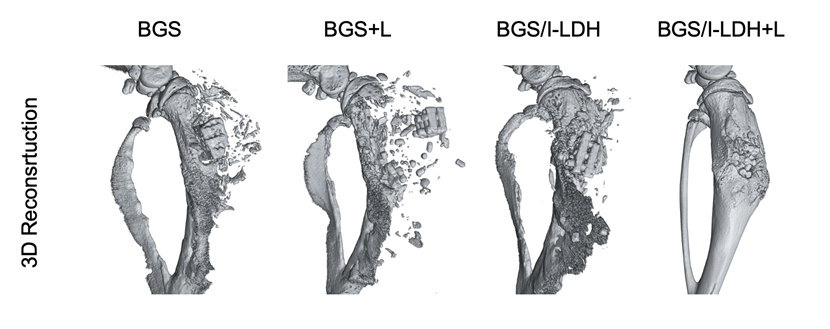


**Figure S30.** 3D micro-CT reconstruction of the tibiae of osteosarcoma-resected nude mice in different groups after sixteen days of scaffold implantation.


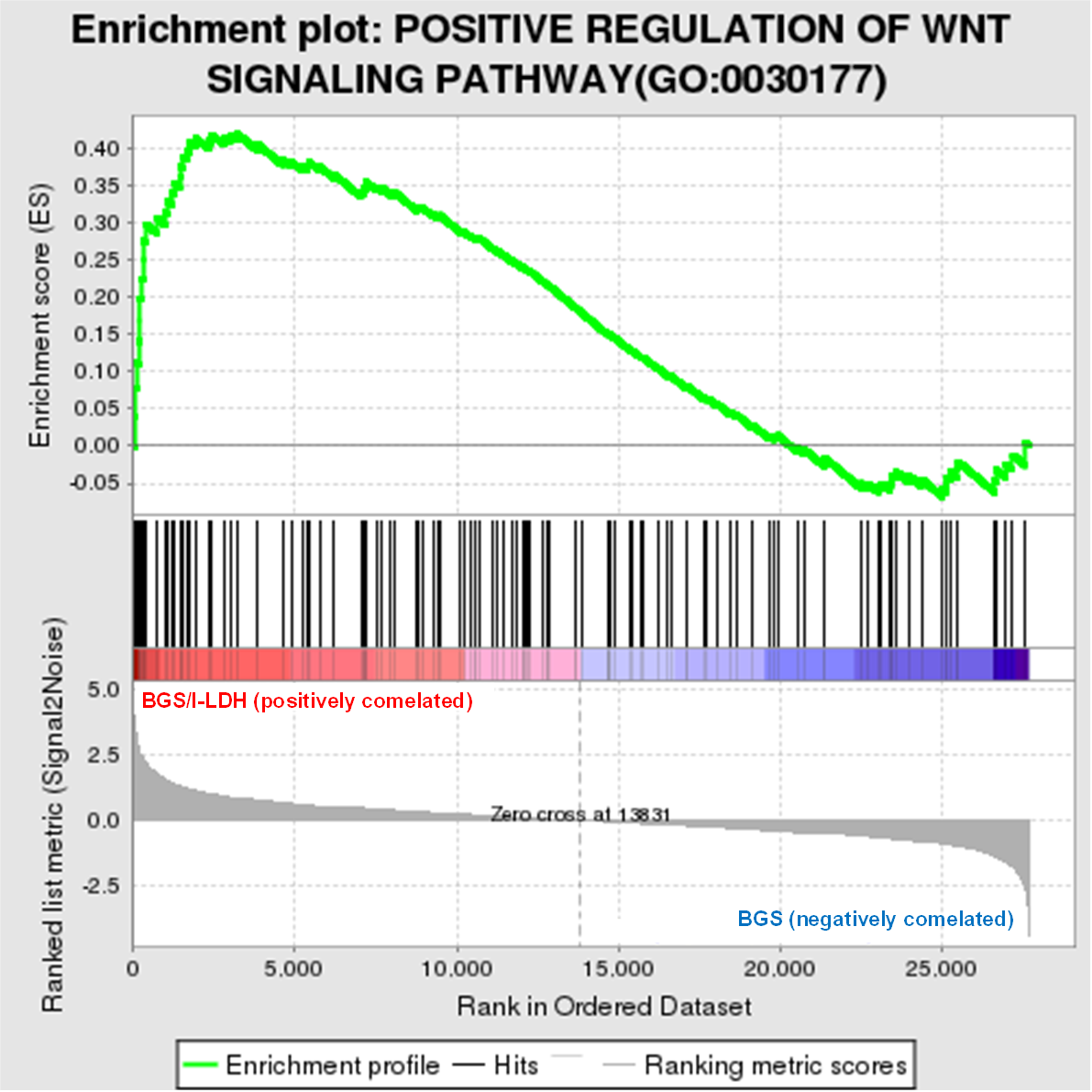


**Figure S31.** Gene Set Enrichment Analysis (GSEA) analysis revealing Wnt signal pathway activation.

**Figure S32.** qPCR results revealing the expression levels of Wnt1, β-catenin, COL1A1, and COL1A2 in control, BGS, and BGS/I-LDH groups.

**Figure S33.** a) West blot assays determining the protein expression levels of Wnt1, β-catenin, COL1A1, and COL1A2 of hBMSCs in indicated groups, and b) corresponding quantitative analysis. GAPDH was used as a loading control.


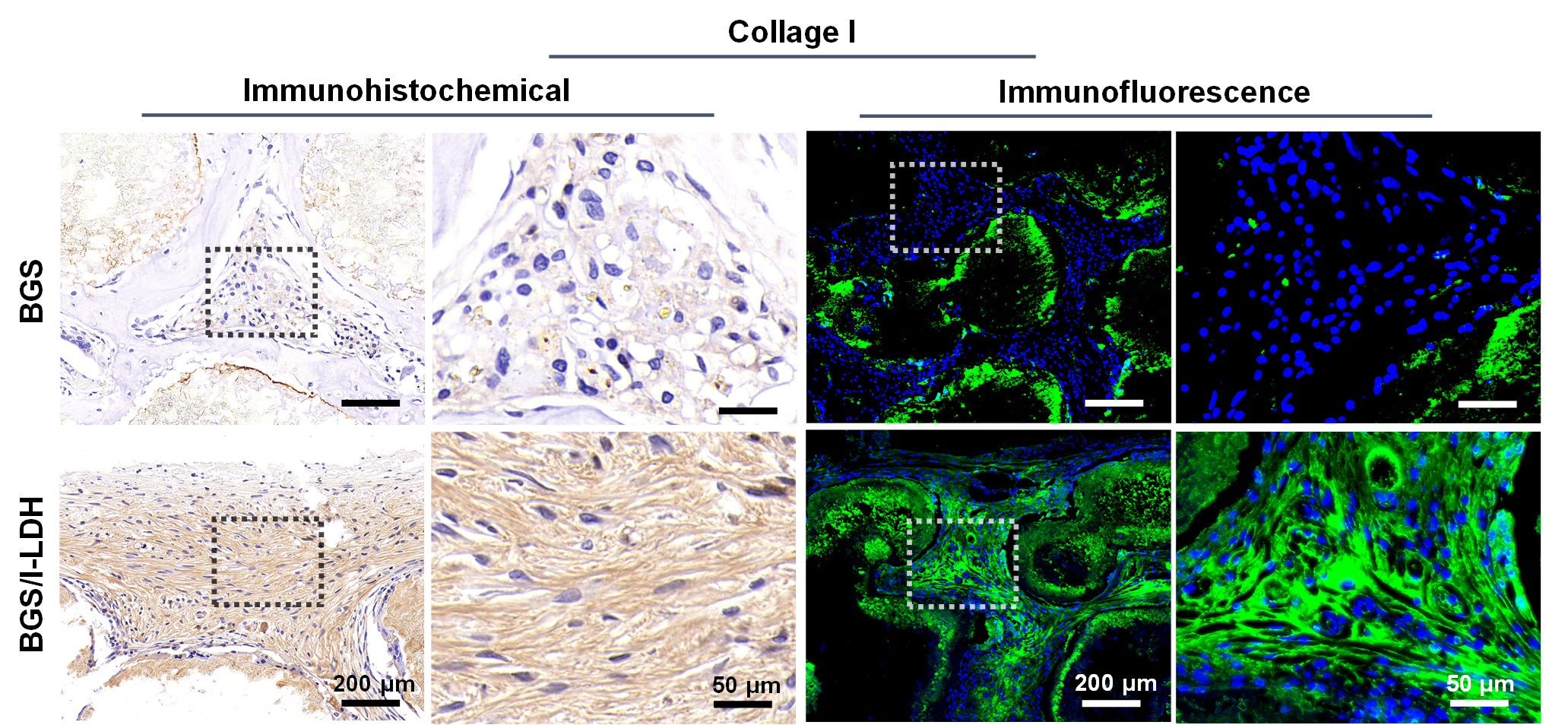


**Figure S34.** Imunohistochemistry and immunofluorescence staining images determining the collage I expression surounding BGS and BGS/I-LDH scaffolds.

**
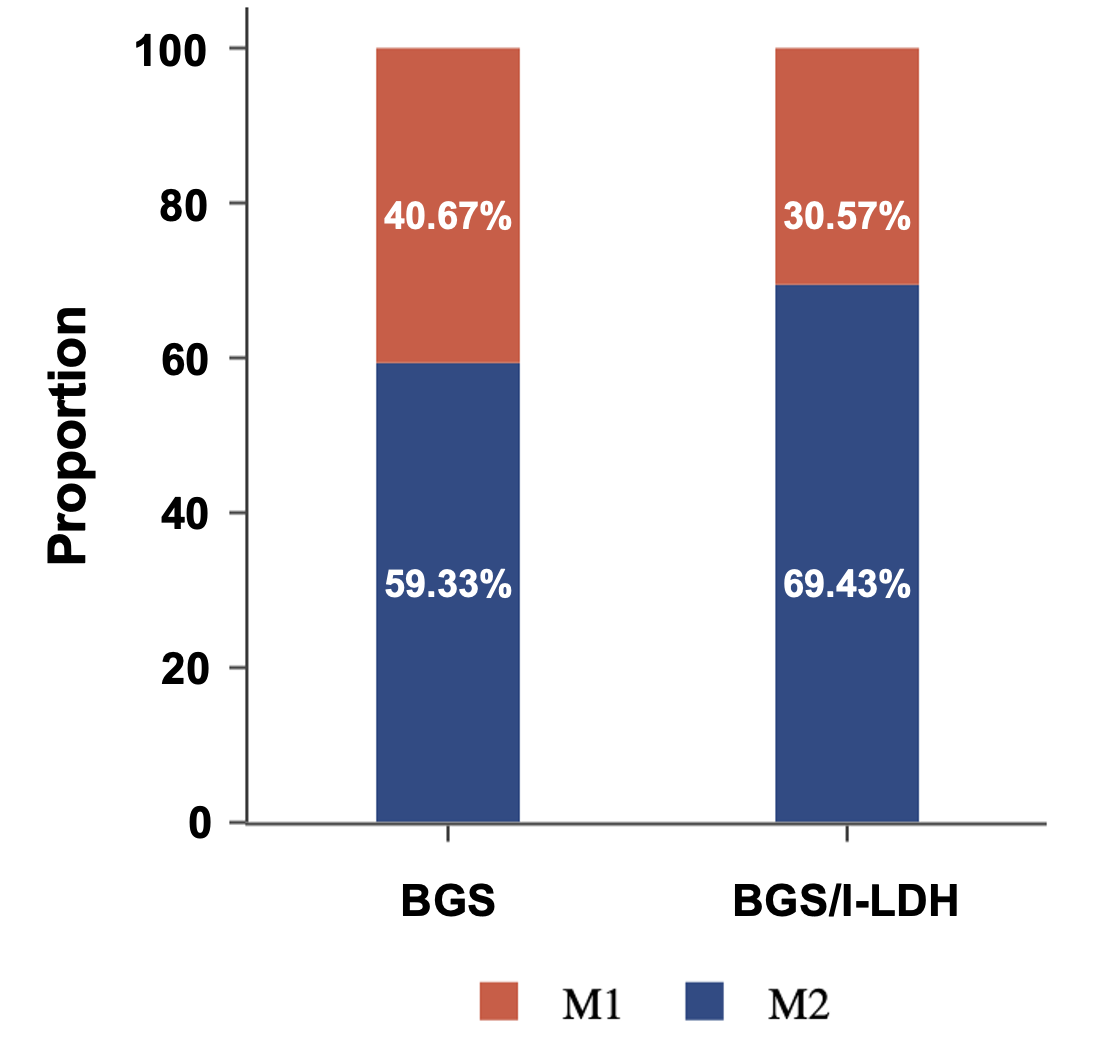
**

**Figure S35.** Sub-clustering analysis of M1 and M2 macrophages in the BGS and BGS/I-LDH groups.

**Table S1.** Ratio of elements in the I-LDH.

| **Sample** | **Element** | | | |
| --- | --- | --- | --- | --- |
|  | Mg | Zn | Al | I |
| I-LDH | 1 | 1.148 | 1.001 | 1.635 |

**Table S2.** Content of elements in the BGS/I-LDH (μmol).

| **Sample** | **Element** | | | |
| --- | --- | --- | --- | --- |
|  | Mg | Zn | Al | I |
| BGS/I-LDH | 1.257 | 1.678 | 1.371 | 1.535 |

**Table S3.** Comparison of the ^1^O_2_ quantum yield of some reported NIR-excited PSs.

| **PSs** | **Reference PSs** | **Laser** | **^1^O_2_ quantum yield** | **Ref.** |
| --- | --- | --- | --- | --- |
| **I-LDH** | **Rose Bengal (RB)** | **1270 nm** | **1.53** | **This work** |
| CoMo-LDH | RB | 1567 nm | 0.875 | [1] |
| CoCuMo-LDH | RB | 1270 nm | 1.06 | [2] |
| PTPEAQ-NP | RB | 400-1000 nm | 0.82 | [3] |
| PTPEAQ-PEG-NP |  |  | 0.34 |  |
| PTPEAQ |  |  | 0.22 |  |
| Tm_2_O_3_@PAA-RGD | Methylene blue (MB) | 1270 nm | 0.36 | [4] |
| DPP-BT NPs | MB | 1270 nm | 0.273 | [5] |
| Au NPN6.5 | MB | 1064 nm | 0.42 | [6] |
| CuO/Cu_2_O TNCs | MB | 1064 nm | 0.24 | [7] |
| EuGdO*_x_*@MSF | MB | 1007 nm | 0.21 | [8] |
|  |  | 948 nm | 0.18 |  |
| UCNP@mSiO_2_-NBCCPT@DHMA/β-CD | MB | 980 nm | 0.45 | [9] |
| NIR-TADF NPs | MB | 880 nm | 0.21 | [10] |
| UF-TTOEH-2Cl | Indocyanine green (ICG) | 880 nm | 0.172 | [11] |
| IrHA2-NPs | ICG | 808 nm | 0.549 | [12] |
| PNDI-2T NPs | ICG | 808 nm | 0.387 | [13] |
| FA-CNPs | ICG | 808 nm | 0.186 | [14] |
| RuDA-NPs | ICG | 808 nm | 0.164 | [15] |
| IrDAD | ICG | 808 nm | 0.146 | [16] |
| BHcy NPs | ICG | 808 nm | 0.129 | [17] |
| IDCIC | ICG | 808 nm | 0.091 | [18] |
| Y16-Pr | ICG | 808 nm | 0.083 | [19] |
| COi6-4Cl NPs | ICG | 808 nm | 0.027 | [20] |
| ETTC | ICG | 808 nm | 0.066 | [21] |
| UPFB | ICG | 808 nm | 0.021 | [22] |
| ICG-NBs-O_2_ | ICG | 808 nm | 0.016 | [23] |
| BTH-Cy7-TCF | ICG | 808 nm | 0.013 | [24] |
| IND-Cy7-TCF |  |  | 0.0019 |  |
| DTPT | IR26 | 808 nm | 0.076 | [25] |
| BODIPY-Br_2_ | Rhodamine B (RhB) | 808 nm | 0.36 | [26] |
| CyI | Cyanine 7 (Cy7) | 808 nm | 0.75 | [27] |
| UCNP@SiO_2_/PPa&DOX@Cs-FA | Pyropheophorbide-a (PPa) | 808 nm | 0.791 | [28] |

**Table S4.** Comparison of the osteogenic properties of some reported modified bioactive glass scaffolds.

| **Scaffolds** | **Bone Volume (Folds)** | **Bone mineral density (Folds)** | **Bone Mass (Folds)** | **Duration (Weeks)** | **Average (Folds/weeks)** | **Ref.** |
| --- | --- | --- | --- | --- | --- | --- |
| **BGS/I-LDH** | **3.8** | **3.0** | **11.4** | **8** | **1.43** | **This work** |
| BGNC@miRNA | ~1.4 | / | / | 4 | / | [29] |
| CBP/MBGS/PTHrP-2 | ~1.5 | ~1.9 | ~2.9 | 8 | 0.36 | [30] |
| FeSAC-BG | ~1.5 | ~1.8 | ~2.7 | 8 | 0.34 | [31] |
| BP-BG | ~1.8 | ~1.3 | ~2.3 | 8 | 0.29 | [32] |
| Se-MBG | ~1.4 | ~1.1 | ~1.5 | 8 | 0.19 | [33] |
| BG-CFS | ~0.9 | ~0.8 | ~0.7 | 8 | 0.09 | [34] |
| CeO_2_–BG | ~1.7 | / | / | 12 | / | [35] |
| Cu-BGC | ~1.0 | / | / | 12 | / | [36] |
| MBS | ~2.0 | ~1.5 | ~3.0 | 16 | 0.19 | [37] |
| NBGS | ~1.5 | ~1.2 | ~1.8 | 24 | 0.08 | [38] |
| Ti_3_C_2_-BG | ~1.3 | ~1.2 | ~1.6 | 24 | 0.06 | [39] |

**References**

1. W. Shen, T. Hu, X. Liu, J. Zha, F. Meng, Z. Wu, Z. Cui, Y. Yang, H. Li, Q. Zhang, L. Gu, R. Liang, C. Tan, *Nat. Commun.* **2022**, *13*, 3384.
2. Y. Yang, T. Hu, Y. Bian, F. Meng, S. Yu, H. Li, Q. Zhang, L. Gu, X. Weng, C. Tan, R. Liang, *Adv. Mater.* **2023**, *35*, 2211205.
3. W. Wu, G. Feng, S. Xu, B. Liu, *Macromolecules* **2016**, *49*, 5017.
4. D. Duosiken, R. Yang, Y. Dai, Z. Marfavi, Q. Lv, H. Li, K. Sun, K. Tao, *J. Am. Chem. Soc.* **2022**, *144*, 2455.
5. Q. Wang, Y. Dai, J. Xu, J. Cai, X. Niu, L. Zhang, R. Chen, Q. Shen, W. Huang, Q. Fan, *Adv. Funct. Mater.* **2019**, *29*, 1901480.
6. N. Kuthala, M. Shanmugam, X. Kong, C.-S. Chiang, K. C. Hwang, *Nanoscale Horiz.* **2022**, *7*, 589.
7. M. Shanmugam, N. Kuthala, R. Vankayala, C.-S. Chiang, X. Kong, K. C. Hwang, *ACS Nano* **2021**, *15*, 14404.
8. P. Kalluru, R. Vankayala, C.-S. Chiang, K. C. Hwang, *Adv. Funct. Mater.* **2016**, *26*, 7908.
9. X. Yao, M. Li, B. Li, C. Xue, K. Cai, Y. Zhao, Z. Luo, *Chem. Eng. J.* **2020**, *390*, 124516.
10. F. Fang, Y. Yuan, Y. Wan, J. Li, Y. Song, W.-C. Chen, D. Zhao, Y. Chi, M. Li, C.-S. Lee, J. Zhang, *Small* **2022**, *18*, 2106215.
11. L. Zhao, M. Chang, Z. He, Y. Zhao, J. Wang, Y. Lu, *ACS Appl. Polym. Mater.* **2023**, *5*, 1530.
12. J. Zhao, Y. Gao, R. Huang, C. Chi, Y. Sun, G. Xu, X.-H. Xia, S. Gou, *J. Am. Chem. Soc.* **2023**, *145*, 11633.
13. K. Wen, X. Xu, J. Chen, L. Lv, L. Wu, Y. Hu, X. Wu, G. Liu, A. Peng, H. Huang, *ACS Appl. Mater. Interfaces* **2019**, *11*, 17884.
14. Z. He, L. Zhao, Q. Zhang, M. Chang, C. Li, H. Zhang, Y. Lu, Y. Chen, *Adv. Funct. Mater.* **2020**, *30*, 1910301.
15. G. Xu, C. Li, C. Chi, L. Wu, Y. Sun, J. Zhao, X.-H. Xia, S. Gou, *Nat. Commun.* **2022**, *13*, 3064.
16. J. Zhao, K. Yan, G. Xu, X. Liu, Q. Zhao, C. Xu, S. Gou, *Adv. Funct. Mater.* **2021**, *31*, 2008325.
17. W. Cao, Y. Zhu, F. Wu, Y. Tian, Z. Chen, W. Xu, S. Liu, T. Liu, H. Xiong, *Small* **2022**, *18*, 2204851.
18. B. Li, Y. Gan, K. Yang, E. Pang, X. Ren, S. Zhao, D. He, F. Zhao, B. Wang, P. Yin, X. Song, M. Lan, *Sci. China Mater.* **2023**, *66*, 385.
19. K. Yang, F. Long, W. Liu, Z. Zhang, S. Zhao, B. Wang, Y. Zou, M. Lan, J. Yuan, X. Song, C. Lin, *ACS Appl. Mater. Interfaces* **2022**, *14*, 18043.
20. L. Li, C. Shao, T. Liu, Z. Chao, H. Chen, F. Xiao, H. He, Z. Wei, Y. Zhu, H. Wang, X. Zhang, Y. Wen, B. Yang, F. He, L. Tian, *Adv. Mater.* **2020**, *32*, 2003471.
21. X. Li, F. Fang, B. Sun, C. Yin, J. Tan, Y. Wan, J. Zhang, P. Sun, Q. Fan, P. Wang, S. Li, C.-S. Lee, *Nanoscale Horiz.* **2021**, *6*, 177.
22. C. T. Perciani, L. Y. Liu, L. Wood, S. A. MacParland, *ACS Nano* **2021**, *15*, 7.
23. L. Yang, B. Huang, S. Hu, Y. An, J. Sheng, Y. Li, Y. Wang, N. Gu, *Nano Research* **2022**, *15*, 4285.
24. M. Liang, X. Mu, Y. Li, Y. Tan, X. Hao, Y. Tang, Z. Wang, W. Feng, Y. Lu, X. Zhou, *Adv. Funct. Mater.* **2023**, *33*, 2302112.
25. Q. Wang, X. Niu, L. Yang, J. Liu, J. Wang, X. Xu, W. Tang, W. Huang, Q. Fan, *Mater. Chem. Front.* **2021**, *5*, 5689.
26. L. Liu, L. Fu, T. Jing, Z. Ruan, L. Yan, *ACS Appl. Mater. Interfaces* **2016**, *8*, 8980.
27. J. Cao, J. Chi, J. Xia, Y. Zhang, S. Han, Y. Sun, *ACS Appl. Mater. Interfaces* **2019**, *11*, 25720.
28. X. Wu, Y. Zhang, Z. Wang, J. Wu, R. Yan, C. Guo, Y. Jin, *ACS Applied Bio Materials* **2020**, *3*, 5813.
29. Y. Xue, Y. Guo, M. Yu, M. Wang, P. X. Ma, B. Lei, *Adv. Healthcare Mater.* **2017**, *6*, 1700630.
30. S. Liu, Z. Han, J.-N. Hao, D. Zhang, X. Li, Y. Cao, J. Huang, Y. Li, *Bioact. Mater.* **2023**, *26*, 1.
31. L. Wang, Q. Yang, M. Huo, D. Lu, Y. Gao, Y. Chen, H. Xu, *Adv. Mater.* **2021**, *33*, 2100150.
32. B. Yang, J. Yin, Y. Chen, S. Pan, H. Yao, Y. Gao, J. Shi, *Adv. Mater.* **2018**, *30*, 1705611.
33. D. Chen, Z. Liang, Z. Su, J. Huang, Y. Pi, Y. Ouyang, T. Luo, L. Guo, *ACS Appl. Mater. Interfaces* **2023**, *15*, 34378.
34. W. Dang, T. Li, B. Li, H. Ma, D. Zhai, X. Wang, J. Chang, Y. Xiao, J. Wang, C. Wu, *Biomaterials* **2018**, *160*, 92.
35. M. Zhang, X. Zhai, T. Ma, Y. Huang, M. Jin, H. Yang, H. Fu, S. Zhang, T. Sun, X. Jin, Y. Du, C.-H. Yan, *ACS Nano* **2023**, *17*, 4433.
36. R. Lin, C. Deng, X. Li, Y. Liu, M. Zhang, C. Qin, Q. Yao, L. Wang, C. Wu, *Theranostics* **2019**, *9*, 6300.
37. Q. Yang, H. Yin, T. Xu, D. Zhu, J. Yin, Y. Chen, X. Yu, J. Gao, C. Zhang, Y. Chen, Y. Gao, *Small* **2020**, *16*, 1906814.
38. J. Yin, S. Pan, X. Guo, Y. Gao, D. Zhu, Q. Yang, J. Gao, C. Zhang, Y. Chen, *Nano-Micro Letters* **2021**, *13*, 30.
39. S. Pan, J. Yin, L. Yu, C. Zhang, Y. Zhu, Y. Gao, Y. Chen, *Adv. Sci.* **2020**, *7*, 1901511.
